# Ethical reasoning and participatory approach towards achieving regulatory processes for animal-visitor interactions (AVIs) in South Africa

**DOI:** 10.1371/journal.pone.0282507

**Published:** 2023-03-06

**Authors:** Alessia Muzzo, Ilaria Pollastri, Pierfrancesco Biasetti, Gregory Vogt, Raoul Manenti, Barbara de Mori

**Affiliations:** 1 Department of Comparative Biomedicine and Food Science, University of Padova, Legnaro, Padova, Italy; 2 Ethics Laboratory for Veterinary Medicine, Conservation, and Animal Welfare, University of Padova, Legnaro, Padova, Italy; 3 Department of Reproduction Management, Leibniz Institute for Zoo and Wildlife Research, Berlin, Germany; 4 Conservation Guardians, Shongweni Nature Reserve, Kwa Zulu Natal, Outer West Durban, South Africa; 5 Department of Environmental Science and Policy, University of Milan, Milano, Italy; Hackensack Meridian Health, UNITED STATES

## Abstract

South Africa’s wide range of animal facilities offers many different types of Animal-Visitor Interactions, wild animal encounters where animals and visitors come closer than in normal circumstances. The aim of this study was to provide a map of the ethically relevant aspects involved in AVIs in South Africa as a first step towards regulating these activities. A participative approach based on the ethical matrix, a tool which organizes the ethical standings of the stakeholders by three bearing ethical principles (wellbeing, autonomy, fairness), was applied. The matrix was populated through a top-down approach and refined by engaging stakeholders in a workshop and two online self-administrated surveys. The outcome is a map of the value demands concerning Animal Visitor Interactions. This map shows how the ethical acceptability of AVIs is linked to different relevant issues like animal welfare, education, biodiversity conservation, sustainability, human competency, facility mission, impact on scientific research and socio-economic outcomes. In addition, results highlighted the importance of cooperation among stakeholders and suggested that attention for animal welfare can inform decision making and inspire a multidisciplinary approach in implementing a regulatory frame for South African wildlife facilities.

## 1. Introduction

Wild animal encounters are increasingly popular activities offered to visitors by a variety of organizations and facilities: from zoos, aquaria, and sanctuaries proposing encounters with program or ambassador animals [1] to the nature-based tourism industry providing activities with free-ranging animals or in dedicated premises [2, 3]. Some of these activities involve Animal-Visitor Interactions (AVIs)—that is, activities where visitors encounter the animals at a distance closer than allowed in usual circumstances [1, 4]. These activities may include: low proximity AVIs where the experience, while still closer than usual, is mediated by a barrier of some sort (behind the scenes encounters, animal shows, etc.); medium proximity AVIs where visitors may experience close proximity without barriers, but with a relatively low expectation of direct contact (non-hand feeding, walkthrough or swim-through, etc.); and close proximity AVIs, where direct contact is an expected and essential part of the activity (touch-pools, direct animal feeding, tactile encounters, petting, animal riding, walk-with or swim-with activities, etc.). In any case, AVIs do not include behaviors that are not allowed but result in interaction (e.g., tank banging).

AVIs can impact human wellbeing (both of visitors and caregivers), animal wellbeing and welfare, and biodiversity conservation [5], with effects that may range from positive to neutral or negative [6–8]. Understanding the implications of AVIs on animal welfare, conservation, and people is a challenging and an emerging field of research [9, 10]. This field of research is complicated by the many variables involved—the type of AVI, the species involved, the individual characteristics of the animals and their position on the wild-captive continuum, the type of facility, the management practices occurring, etc. For this reason, AVIs need to be investigated also from an ethical standpoint, taking into account the different value dimensions relative to respect for people, animals and biodiversity, and the way they relate with each other [9, 11].

The need for a multidimensional ethical analysis of AVIs is made even more urgent by the proliferation of these activities, which goes along with the need of achieving a regulatory approach. Globally, wildlife tourism is a growing industry, and the possibility of interacting with animals provides great attractiveness. In this global context, South African wildlife tourism facilities may offer to their guests one of the greatest range of activities—giraffe-feeding, interactions with semi-captive elephants, lion and cheetah walks, snake demonstrations, meerkat interactions, carnivores-feeding shows, cub-petting, and so on. While AVIs require that complex trade-offs between profitability, animal welfare, and species conservation be made, if responsibly managed, they are conceived to be able to provide important opportunities for the local economy, biodiversity conservation, visitor education, and also for animal welfare [10]. At the same time, however, poor management can bring animal welfare, conservation, and economic sustainability into direct collision [10].

This study aimed to provide a map of the ethically relevant aspects involved in AVIs in South Africa as a possible first step towards regulating these activities. To consider a wide range of perspectives and include into the analysis the contextual variables from the South African scenario, a participative approach based on ethical reasoning was adopted. A workshop was organized and two online surveys were subsequently launched to build an Ethical Matrix (EM). The EM is a conceptual tool for conducting structured ethical analysis on existing or prospective technologies, situations, dynamics, and policy options, and to support decision-making [12, 13]. It is not a prescriptive tool [14] but helps decision-makers in reaching responsible and defensible decisions [13] by summarizing the moral interests involved, pointing out the eventual conflicts, and anticipating the positive and negative impacts on the stakeholders of the issue under investigation. It was introduced in the literature by Ben Mepham in the context of food ethics [15] and it has since been applied to several fields including forestry [16], fishery technology [17, 18], radiation restoration strategies [19, 20], conservation practices and policies [21–23], as well as in the assessment of human-animal interactions [4, 24, 25]. The opportunities provided by structuring a participatory process through EM are well known [26, 27]. In particular, the use of the EM encourages the participants to take into consideration the others’ perspectives, allowing in this way—as much as possible—for a plural and comprehensive collection of the ethically relevant aspects. In this study, a customized EM was created by collecting data through a participative process to be used as a first step toward regulating AVIs in South Africa.

## 2. Materials and methods

The study took place between November 2019 and December 2020 and consisted of building an EM by collecting the ethically relevant demands involved in AVIs in South Africa through a participatory process.

During the first phase of the study, stakeholders were defined following Mepham et al. (2006) [13]. Either interest groups (human or not human) “actively affecting” or “affected by” the issue were included. The proposals of the research group members were integrated into a brainstorming group, during which the final list was defined. The list included: a) animals involved in AVI; b) owners and managers; c) handlers; d) keepers and staff; e) veterinarians; f) government representatives; g) biodiversity; h) visitors participating in AVIs; i) animal rights groups. The EM was then sketched top-down by the members of the research group, using scientific and grey literature on the topic [4, 12–14, 16–18, 21–29]. Subsequently, this first draft underwent a bottom-up process of refinement. During this second step of the study, data collected in a participatory process—a one-day facilitated workshop and two online surveys—were organized and analyzed, and were then used, along supplementary scientific and gray literature, to build the detailed Final EM for AVIs. The outcome of the EM was then revised top-down, and multiple brainstorming sessions and revision phases allowed to define the concepts representing the stakeholders’ interests, a draft report was prepared and distributed amongst participant stakeholders to obtain final feedbacks. Finally, a final report, including relevant data and the Final EM for AVIs was then completed and sent to the government representatives as a first step towards regulating AVIs in South Africa.

The study was performed in compliance with the relevant ethical and normative guidelines of South Africa. No approval of an ethics committee/institutional board was needed ate the time of the study. Workshop participants voluntarily joined the study and gave their oral consent for inclusion before participating. Participants were assured of anonymity unless specific requests for the contrary, and no personal information was collected. Survey respondents gave their informed consent for inclusion. A privacy notice was provided at the beginning of the survey to inform and assure that responses were anonymous and confidential and that information collected would be used for research purposes only. No personal information was collected, and only visitors over 18 years old could participate. Participation was voluntary and could be canceled at any time without any reason. No incentive or financial reimbursement was provided.

### 2.1. Step 1: The participatory process

After sketching an *interim* EM top-down based on the relevant literature (S1 Table) and identifying the relevant stakeholders to be contacted, a participatory process involving a one-day workshop and two surveys was carried out to collect data. Data collected were then used to refine the EM top-down. The main goals of the participatory process was to ensure that stakeholders could personally advance and discuss their ethically relevant interests, and, at the same time, identify and discuss the interests of animals and biodiversity. Through the participatory process it was possible to collect data specifically to: (a) cross-check and confront the value-demands at stake; (b) assess the importance attributed by the stakeholders to the various value-demands identified.

#### 2.1.1. The workshop

Workshops are part of the standard methodology of the bottom up EM [13]. In this case, a one-day facilitated workshop, hosted by Shongweni Dam and Nature Reserve NPC (29°51’35”S 30°43’20”E) was organized on November 20^th^, 2019 in partnership with Conservation Guardians (www.conservationguardians.africa), who took care also of involving participants.

Relevant stakeholders (i.e. affected parties identified by the sketched EM) as potential participants were contacted. The invitation was sent by e-mail to 12 Facilities/Organizations/ Institutions, then followed up by phone call. Nine of Facilities/Organization/Institutions attended the workshop with one or more representatives, for a total of 18 invited participants. Their professions were: owners and managers of facilities (n = 9); keepers (n = 2); government representatives (n = 2); wildlife veterinarians (n = 1); and academic researchers (n = 4). Owner and managers came from game farms, safari parks, zoos, aquaria, and facilities hosting elephants or lions. Their professional backgrounds included conservationists, animal welfare and behavior experts, field rangers, high-level keepers, and trainers.

The workshop was co-facilitated by University of Padova and Conservation Guardians members. Two researchers were tasked with taking minutes of the workshop (as suggested by [18]), preparing visual contents to support the process, and checking the logistical aspects. Audio recording of the workshop also took place, after written consent was given by all participants.

The workload was divided into four stages: opening, preliminary session, main session, and closure.

*Opening of the workshop*. The opening consisted of an introduction on the aims of the workshop, on ethics, and the EM. An operative definition and classification of AVIs was also discussed in this phase.

*Preliminary session of the workshop*. The preliminary session included a 1^st^ round and a 2^nd^ round. During the 1^st^ round of the preliminary session, blocks of sticky notes were distributed among participants, who were asked to identify key animal welfare issues and key management issues concerning AVIs by writing them down [30]. Only one issue could be written on a single note, and no fixed limit to the number of notes that could be used was given. All sticky notes were then collected and displayed on a board; animal welfare issues on one side, management issues on the other. Each sticker was tagged with a pre-assigned numeric code, specifically assigned to each participant (as per [31]). This permitted researchers to identify the author of each note while assuring anonymity among participants, and minimizing the influence that they could have on each other.

Afterwards, researchers grouped the notes with similar themes, and assisted by the facilitators, assigned a temporary title to each cluster. Clusters and titles were then discussed with participants. Participants were invited to debate, agree or amend the composition of clusters and the temporary titles. During the discussion clusters were added to form larger grouping, others were instead split, and notes were moved from one cluster to another.

Once an agreement on clusters and title was reached, the 2^nd^ round started. The goal of this round was to identify the perceived priorities of the participants amongst the clusters. Each participant voted three animal welfare clusters and three management issues clusters as her or his priority. Sticky notes were used for voting, and, after collection and counting, the six most voted clusters for each category were displayed on the board, animal welfare clusters on one side, management issues clusters on the other. The results provided a starting point for main session activities.

*Main session of the workshop*. During the main session, stakeholders were asked to advance their value demands. The basic structure of the EM was briefly recapped, and an empty matrix was displayed. Participants were asked to individually express, using sticky notes, their opinion on the necessary criteria for their wellbeing, autonomy, and fair treatment, also referring to the notes individuated in the previous step and still present on the board. During the entire exercise, facilitators were available to assist participants and give them further information. All participants then attached their stickers to the empty cells of the EM. An open discussion followed.

*Closure of the workshop*. During the closure phase, anonymous feedback from participants was collected using a questionnaire to support the SWOT analysis (Analysis of Strengths, Weaknesses, Opportunities, Threats) [32–34].

#### 2.1.2. SWOT analysis

As recommended [13], a SWOT analysis [32–34] was performed to evaluate strengths, weaknesses, opportunities, and threats of the workshop experience. The SWOT analysis focused on the methodology and aims of the workshop. The feedback form distributed was anonymous and participation voluntary. There was no time limit to complete the questionnaire and a researcher collected the filled forms one by one.

#### 2.1.3. The surveys

The use of surveys introduced an element of novelty in the standard methodology of the EM. They were adopted to include the point of view of stakeholders that were difficult to involve in the workshop activities (i.e., visitors of facilities), and were necessary to be represented into the EM in order to follow criteria of inclusivity and completeness.

Two different surveys were specifically designed, one aimed at the staff of facilities and another at visitors. Both surveys aimed to investigate the value demands of the respondents, in order to identify their perceived criteria for their wellbeing, autonomy, and fair treatment. Moreover, the staff questionnaire also investigated the staff perspective on animal welfare and management issues related to AVIs, similarly to what was done during the preliminary session with stakeholders participants to the workshop.

Google Forms, a user-friendly web-based tool, was used to create and conduct the two online surveys, which were based on an anonymous self-administrated questionnaire. The surveys were set up using convenience sampling, also known as Haphazard Sampling or Accidental Sampling, a type of nonprobability sampling where members of the target population meet certain practical criteria [35]. In this study, such criteria were the accessibility and the willingness of the respondents to participate in the study. Due to privacy reasons, it was not possible to directly access visitors and staff emails. Therefore, facilities taking part in the study submitted the survey link to their past visitors—the ones who gave consent to the facility to use their email contacts—and to their Staff (keepers, educators, handlers, etc.).

The questionnaires were reviewed and pilot tested to identify confusing items, mistakes, and potential biases [36] by a small group of experts and not-experts, who were asked to complete the form and report what they found easy or difficult to understand, confusing and interesting. No data was analyzed in this phase, and the feedbacks were exclusively used to refine and finalize the questionnaires.

Data collection for the visitor survey began in April 2020 and continued until December 2020. The visitor questionnaire consisted of three sections: 1) “Demographical Section”; 2) “PV Section”, for Participating Visitors (PV)—visitors who experienced AVI; 3) “NPV section” for Non-Participating Visitors (NPV)—visitors who did not experience any AVI (the visitor questionnaire is available in S2 Table).

#### Demographical section

The demographical Section consisted of six items and included questions about the age, nationality, and gender of respondents, as well as on their self-perception and the period of the visit to the facility. At the end of the section, respondents were asked if they had experienced AVIs during their visit and were directed to the “PV Section” or the “NPV section” according to their answers.

#### PV section

The PV Section consisted of 15 items and included questions on the AVI experienced by the visitors (AVI description, questions n. 7–8), on their criteria for their well-being (questions n. 9–14), autonomy (questions n. 15–17), and fair treatment (questions n. 18–19), and general feedback and additional comments (questions n. 20–21).

#### NPV section

NPV Section included two questions, one asking why the respondent did not experience AVIs (question n. 22), the other collecting additional comments (question n. 23).

Data collection for the staff survey began in July 2020 and continued until December 2020. The staff questionnaire consisted of four sections: 1) “Preliminary Information Section”, to allow redirection to either “AVI Section” or “No AVI Section”; 2) “AVI Section”, dedicated to staff in care of animals involved in AVIs; 3) “No AVI Section”, dedicate to staff in care of animals not involved in AVIs; and 4) “Staff Demographics Section” (the staff questionnaire is available in S3 Table).

#### Preliminary information section

Questions in this section recorded the facility in which the respondent was working and sorted staff caring for animals involved in AVIs from staff not involved (questions n. 1–2).

#### AVI section

Members of the staff caring for animals involved with AVIs were directed here from the preliminary information section. AVI section included six subsections, each with its specific goal: (a) to collect details about the AVIs and the animals under the responsibility and care of the respondent; (b) to collect their criteria for their wellbeing, autonomy and fair treatment, using five-points Likert scale (questions n. 4–16); (c) to identify three main animal welfare issues concerning AVIs and possible solutions or mitigation strategies (questions 17–18); (d) to identify three key management issues concerning AVIs and possible solutions and mitigation strategies (questions 19–20); (e) to investigate safety perception, by asking the respondents to indicate how often they feel unsafe during their work with animals, what are the main dangers concerning AVIs, and their suggestions on how to improve safety (questions n. 21–23); f) to get feedback, discover if, in the last year, the staff was involved in any meeting to promote animal welfare, conservation strategies, and educational activities for the visitors, and to collect suggestions on how to improve AVIs (questions n. 24–25).

#### No AVI section

Members of the staff not caring for animals involved with AVIs were directed here from the preliminary information section. No AVIs section included three subsections, each with its specific goal: (a) to collect details about the animals under the responsibility and care of the respondent (question n. 35); (b) to collect their criteria for their wellbeing, autonomy and fair treatment, using five-points Likert scale (questions n. 36–48); c) to get feedback, discover if, in the last year, the staff was involved in any meeting to promote animal welfare, conservation strategies, and educational activities for the visitors, and to collect suggestions on how to improve AVIs (questions n. 49–50).

#### Staff demographics section

This section grouped a wide range of demographical questions (questions n. 26–34 “AVI Section”; questions 51–59 “No AVI Section”.

### 2.2 Step 2: Final EM for AVIs

During the second step of the study, data collected in the participatory process (workshop and surveys) were organized and analyzed, and were then used, along supplementary scientific and gray literature, to build the detailed *Final EM* for AVIs. Stakeholders’ interests were defined during multiple brainstorming sessions and revision phases and reported in the Final EM for AVIs. S2 and S3 Tables summarize the link between the survey questions and the value demands of the respondents, and the staff perspective on animal welfare and management issues related to AVIs.

#### 2.2.1. Data analysis—workshop

Materials from the workshop were checked, notes were associated with the correspondent participant codes, votes were screened, ranking of animal welfare and management clusters was performed. Issues and clusters were then analyzed according to authorship, to identify the preferences of each stakeholder.

Minutes, notes on the key discussion point, and audio recordings of the workshop were then used to craft a report. The draft report included the list of AVI animal welfare and management issues brought up by the participants; the thematic clusters into which they were collected; the ranking of the clusters; the notes of the discussion on the EM. After a first revision by the facilitators, the draft report was sent to all the workshop participants, inviting them to contribute, comment and revise. A final report was then prepared, including stakeholders’ comments and revisions and the final EM built on the basis of data collected both from the workshop and the surveys, and sent to the government representatives.

#### 2.2.2. Data analysis—surveys

Different statistical analyses were performed to understand the eventual impact of socio-cultural factors, time passed from the experience, and demographical factors (age, gender, etc.) on the perception and interests of visitors regarding AVIs. After descriptive analyses on data collected, a series of linear mixed effects models (LMMs) and generalized mixed effects models (GLMMs) were generated using as dependent variables: (a) the level of satisfaction with the experience of the respondents (question n. 9); (b) the level of safety perceived by the respondents (question n. 10); (c) the final profile of respondents (Amusement, Education, Emotion or Neutral—question n. 11–13; binomial error distribution); (d) the mindset of respondents (Animal-centric, Biodiversity-centric, Ethics-centric—question n. 14; binomial error distribution); and the economical affordability perceived by respondents (question n. 18). In each model generated, the following independent variables were included: (a) age; (b) gender; (c) self-definition of respondents; (d) and time passed from the visit. In the models in which final profiles and mindsets of respondents were not considered as dependent variables, they were added as independent factors. The facility in which the respondents performed their interactions was included as a random factor. After model building, the significance of the independent variables composing each model was assessed using Wald F and χ2 tests [37].

Questions n. 11, 12, 13 were analyzed both as separate questions and as a conjoint subset of questions to generate a “final profile” for each respondent. This final profile represents what the respondent prioritizes during the AVI experience between “need of amusement”, “need of education” and “need to be emotionally close to animals”. According to the answers to questions n. 11–13, each respondent was assigned to one of the four possible final profiles: “Amusement”, “Education”, “Emotion” or “Neutral”. Respondents who showed prevalent interest in education, being emotionally close to animals or a prevalent attitude towards amusement in two or more of the answers were assigned to the final profile Education, Emotion, or Amusement, respectively. Respondents were classified Neutral if they choose one answer per type in the three questions (“need of amusement”, “need of education”, “need to be emotionally close to animals”).

Question n. 14 aimed to identify which aspects of education were more important in the mindset of visitors among the proposed answers. Depending on what they prioritized between “learning about animals”, “biodiversity and conservation”, or “learning about the origin and welfare of the animals hosted in the facility, its mission, and the captive-related problems”, respondents were classified to have an "animal-centric", "biodiversity centric" or "ethics-centric" mindset respectively.

Answers to open question n. 19 and n. 21 were studied and summarized in the results. To facilitate question n. 19 analysis, two different researchers independently assigned tags to each item in the answers (maximum six items per respondent) and then grouped them in broader categories. Afterwards, the work of the two researchers were compared, tags and grouping were reviewed and a final analysis was elaborated. This procedure was not meant to obtain quantitative data, but just to implement an effective summarizing process and improve its reliability.

The staff survey was analyzed with descriptive statistics.

All the analyses were performed in R 3.3 environment using the software packages car, LmerTest and glht (R Development Core 2018).

## 3 Results

### 3.1. Workshop results

In total, the 18 participants identified 76 animal welfare issues on the sticky notes (with a mean of 4.22 animal welfare issues per participant). After discussing the preliminary categorization proposed by the researchers, participants agreed to define 17 animal welfare clusters related to AVIs. During the 2^nd^ round, participants indicated (with three votes each) the animal welfare clusters, which, in their opinion, should be prioritized. The most voted cluster was *Human competency* (8 votes), followed by *Best practice*, *Compliance*, *Health* (5 votes each), and *Animal rights interference* and *Safety (Animal*, *Human*) (4 votes each). Table 1 presents the Animal Welfare clusters and votes, and S4 Table details identified issues, clusters, and votes.

**Table 1:** Animal welfare clusters and votes (OM = Owners and Manager; R = Researchers; HKS = Handlers/Keepers/Staff; GR = government Representatives; V = veterinarians). Full details in S4 Table.

Animal Welfare Clusters	Stakeholders who identified the issues	Stakeholders who voted for the cluster	Number of votes for cluster
Human competency	OM; V	GR; HKS; OM; R	8
Best practice	GR; R	OM; R; V	5
Compliance	GR; OM	HKS; OM; V	5
Health	OM; R; V	OM; R;	5
Animal rights interference	GR; OM	GR; OM; R	4
Safety (Animal, Human)	GR; OM; R	HKS; OM	4
Assessment (animal)	OM; R; V	OM; R	3
Implementing husbandry	HKS; OM; R	HKS; OM;	3
Regulating "rules"	GR	GR; HKS; OM	3
Training (animal)	HKS; OM; R	HKS; OM	3
Communication	OM; R	GR; OM	2
Population control (management)	HKS; R	GR; R	2
Regulating interactions	OM; R	OM	2
Zoonosis and diseases	OM; R	R	2
Five domains	HKS; OM	OM	1
Space	OM; R	V	1
Enrichment	R		0

In total, the 18 participants wrote 95 management issues on the sticky notes (mean 5.3 specific issues per participant). After discussing the preliminary categorization proposed by the researchers, participants agreed to define 14 management clusters related to AVI. During the 2^nd^ round, participants indicated (with three votes each) the management clusters, which, in their opinion, should be prioritized. The most voted cluster was *Husbandry and care protocol* (7 votes), *Governance*, *Sustainability* (6 votes each), *Conflicting legislatory bodies*, *Legislation* (5 votes each), and *Communication*, *Conservation education*, *Training people* (4 votes each). *Communication* was considered both an animal welfare and a management issue. Table 2 reports the Management clusters and votes, and S5 Table details identified issues, clusters, and votes.

**Table 2:** Management clusters and votes (OM = Owners and Manager; R = Researchers; HKS = Handlers/Keepers/Staff; GR = government Representatives; V = veterinarians). Full details in S5 Table.

Management cluster	Stakeholders who identified the issues	Stakeholders who voted for the cluster	Number of votes for cluster
Husbandry and care protocol	OM; R; GR;	OM; R; HKS; V	7
Governance	OM; GR; V	OM; R; GR; V	6
Sustainability	OM; HKS; GR	OM; R; HKS; GR	6
Conflicting legislatory bodies	OM;	OM; R; HKS; GR	5
Legislation	OM; R; GR;	OM; R; HKS;	5
Communication	OM; HKS;	OM; R	4
Conservation education	OM; R	OM; R	4
Training people	OM; R; HKS; GR	OM; R; GR	4
Human threats	OM; V	OM; R; HKS	3
Brand Reputation	OM;	OM;	2
Conflicting mandates	GR; OM; V	R; GR	2
Safety (Animal and humans)	OM; R	OM	2
Internal codes of conduct	OM; R; GR; V	V	1
Environmental threats	OM; R		0

All the original suggestions proposed by the participants during the discussion and reflecting their interests in terms of wellbeing, autonomy, and fairness during the *Main session of the workshop* are reported in S6 Table. S6 Table was shared with the participants after the workshop, as well as the report of the day, summarizing the activities and the discussions. The workshop participants were invited to provide feedback and reviews, but no additional information were collected in this phase.

### 3.2. SWOT analysis results

Table 3 reports an evaluation of the Internal (strengths and weakness) and External (opportunities and threats) dimensions of the workshop done with a SWOT analysis. SWOT contents were obtained from the feedback questionnaire administered to the participants at the end of the workshop.

**Table 3:** Internal and external dimensions of the workshop.

STRENGTHS	WEAKNESSES
Collecting different ideas, opinions, and perspectives on the topic. Having diverse stakeholders together at the same table for real-time confrontation and group discussion. Approaches and their novelty in this field appreciated by the participants (discovering and filling the Ethical Matrix, methods, materials); All the participants recognized the value and usefulness of the workshop in aiding the discussion on AVI.	Insufficient time for having a deep discussion in the main session and final synthesis. Not all stakeholders being represented at the workshop.
**OPPORTUNITIES**	**THREATS**
Interest in the Ethical Matrix as a new approach to problem-solving in the field of wildlife management. Possibility to integrate the participatory process with preliminary focus groups. Possibility to repeat the experience inviting other stakeholders and/or creating workshops dedicated to more specific topics. Possibility to organize other workshops, inspired by this experience, to aid the discussion on specific themes and develop possible guidelines or deliverables. Integrating the workshop process with the use of surveys.	Difficulty to have all necessary stakeholders at the table at the same time. Risk of «overdiscussing» issues and difficulty to produce an effective, synthetic deliverable. Difficulty in having stakeholders equally represented physically at the workshop (number of participants per stakeholder group). Stakeholders influencing other stakeholders (i.e., influence due to working relationships, influence that good communicators can have on others).

### 3.3.Visitor survey results

A total sample of 177 visitors answered the questionnaires, n = 19 (11%) from facility A, n = 150 (85%) from facility B, and n = 8 (5%) from facility C. S7 Table summarizes demographic information and other independent variables collected from visitors who answered the questionnaires.

Only 4 out of 177 respondents did not experience AVI, for miscellaneous reasons, while 173 (98%) of the respondents experienced AVI with the elephants of facilities A, B, or C. The following results represent the subset of 173 respondents who experienced AVI.

The 94% (n = 163) of respondents was “extremely happy” with the AVI experience, and the 6% (n = 10) scored 4, so was “happy” with the experience (mean = 4.94, median = 5.00, mode = 5.00). When asked the safety perception during AVI, respondents declared to have a high safety perception: 94% (n = 163) felt “extremely safe”, 5% (n = 9) felt “safe” and only 1% (n = 1) of the respondents felt “neither safe nor unsafe” (score 3) (mean = 4.93, median = 5.00, mode = 5.00).

Questions n. 11–13 investigated what respondents prioritized among three different needs. Answers were tagged according to the “Need of amusement”, “Need of education” and “Need to be emotionally close to animals”. When asked why they decided to participate in the activity (question n.11), most of the respondents fell into the “Need of education” category (76%, n = 132). When asked what they were looking for when participating in the activity (question n.12) and what impressed them the most (question n.13), the majority fell into the “Need to be emotionally close to animals (61%, n = 106 and 52% n = 90 respectively). S8 Table presents the detailed results for each question.

According to the prevailing answers to questions n. 11, 12, 13, each respondent was assigned to an overall “final profile” among “Need of amusement”, “Need of education”, “Need to be emotionally close”, and “Neutral”. “Neutral” final profile was assigned to respondents who presented equally distributed answer types. Final profiles of respondents resulted distributed as follows: 53% (n = 91) “Need of Education”, 42% (n = 72) “Need to Be emotionally close”, 1% (n = 2) “Need of amusement”, and 5% (n = 8) “Neutral”.

Respondents who defined themselves as “Thrill seekers” in 100% of the cases showed a “Need to be emotionally close” profile. The “Need to be emotionally close” profile was also shown by 60% of “Curious tourists” respondents. The ones describing themselves as “Animal experts” or “Animal lover” more frequently had a “Need of Education” profile (82% and 51% respectively), and just 8% of the “Nature lovers” demonstrated a “Neutral” profile. The level of satisfaction with the experience was significantly lower in respondents with the “Need of amusement” final profile (F = 7.51, p<0.01; S9 Table). From the analysis, it also emerges that respondents defining themselves as “Animal experts” or as professionals working with animals and the environment have a significantly higher probability to appreciate education (final profile “Need of Education”; χ^2^ = 5.44, p = 0.01; S9 Table).

Question n. 14 explored the specific interests of respondents concerning the learning opportunities offered by AVIs. Potentially, these experiences can stimulate the curiosity of visitors in these directions: (a) to learn about the animals involved (anatomy, physiology, ethology, captive animal welfare, husbandry, management, keeper-animal relationship, handler-animal relationship, training); (b) to learn about conservation of the animals involved (rehabilitation reintroduction, species survival plan, current challenges, poaching, conservation sustainability, understand impact); (c) to learn about habitats, biodiversity, and the interrelationship between wildlife and environments; (d) to practice ethical reasoning, investigating the mission statements of the facility, purpose of AVI, origin, history, and life of the animal housed in the facility (why these animals are here?). In this way, question n. 14 investigated whether the visitors approached the AVI experience with an “Animal-centric” (a), “Biodiversity-centric” (b and c), or “Ethics-centric” mindset (d). Respondents distributed as follows: 40% (n = 70) “Animal-centric”, 32% (n = 56) “Biodiversity-centric” and 27% (n = 47) “Ethics-centric”.

The relation between mindset and demographic of respondents is reported in Table 4. A weak significant relationship occurs between older respondents and a Biodiversity-centric attitude (χ^2^ = 5.77, p = 0.01; S9 Table), intended as a preferential interest in learning about nature and biodiversity (i.e., Habitat, interrelationship between wildlife and environment, interdependence, endangered species and relative survival plans, sustainable conservation programs, rehabilitation, and reintroduction programs, poaching, human impact on wildlife).

**Table 4:** Relation between mindset and demographic of respondents.

		Animal-centric	Biodiversity-centric	Ethics-centric
**Age (range in years)**	14–18	100% (1)	0	0
	19–25	44% (4)	22% (2)	33% (3)
	26–34	45% (10)	18% (4)	36% (8)
	35–54	40% (32)	35% (28)	26% (21)
	55–64	33% (14)	40% (17)	28% (12)
	over 64	53% (9)	29% (5)	18% (3)
**Gender**	Female	37% (41)	29% (32)	34% (38)
	Male	47% (29)	39% (24)	15% (9)
**Nationality**	Africa	36% (42)	38% (44)	26% (31)
	Australia	50% (1)	0	50% (1)
	Europe	53% (16)	27% (8)	20% (6)
	North America	50% (10)	15% (3)	35% (7)
	South America	25% (1)	25% (1)	50% (2)
**Self-description**	Animal expert	55% (6)	36% (4)	9% (1)
	Animal lover	39% (34)	26% (23)	34% (30)
	Curious tourist	20% (1)	60% (3)	20% (1)
	Nature lover	45% (28)	37% (23)	18% (11)
	Other	17% (1)	17% (1)	67% (4)
	Thrill seeker	0	100% (2)	0
**Need for…**	Amusement	50% (1)	50% (1)	0
	Education	40% (36)	38% (35)	22% (20)
	Emotion	39% (28)	25% (18)	36% (26)
	Neutral	62% (5)	25% (2)	13% (1)

Question n. 15 aimed to explore if visitors perceived to be provided with a sufficient level of information about the facility, specific information regarding the AVI, mandatory behavioral rules to be respected during the interaction, and information about the welfare of the interacting animals. In the 92% (*n =* 159) of the cases, respondents believed to have received sufficient information about all the four topics. The 6% (*n =* 10) of the sample confirmed to have received enough information for all the topics except for ‘facility’, as they declared that they did not look for information about this theme. The other respondents declared to not have received enough information about the behavioral rules (1%, *n =* 2 of the respondents), or about the welfare of the animal/s they interacted with (1%, *n =* 1). One respondent declared to have not looked for information on all the themes (1%, *n =* 1).

Most respondents acquired information about the facility and its activities from friends/family (46%, n = 79), internet (24%, n = 41) or directly at the facility (20%, n = 34). Other sources of information were hotels and holiday rentals (5%, n = 8), travel agencies (3%, n = 6), tourist centers (1%, n = 2) and others (2%, n = 3).

Understanding how visitors tend to choose an animal facility could give an insight into their needs and priorities. When asked about the reason why they chose the visited facility over other ones offering similar activities, 58% (n = 101) of the respondents reported that the reason had been the awareness around welfare standards offered in that specific facility. The 18% (n = 31) of the respondents said it was the closest facility on their travel route, for the 5% (n = 9) of the respondents the visited facility was the only one they had heard of, 4% (n = 7) chose the facility because of the possibility to do other interesting activities in the same facility, and 2% (n = 4) because of the number of animals/species housed. The remaining 12% (n = 21) provided miscellaneous reasons (recommended, for family/friends reasons, etc.).

The affordability of AVIs was investigated by asking the question “Do you think the price you paid is fair?” (question n. 18) and letting respondents express through five points Likert scale starting from “Extremely unfair” (1) to “Extremely fair” (5). The 77% (n = 133) of respondents considered the price paid “extremely fair”, 16% (n = 28) considered it “fair” and 7% (n = 12) “neither fair nor unfair” (score 3) (mean = 4.72, mode = 5.00).

Question n. 19 aimed to understand which factors visitors would consider important in a hypothetic rating system, that could rate the quality of the animal facilities offering AVIs by asking to indicate three criteria, from the most important to the least important. Respondents wrote 544 criteria, giving 0–6 criteria each. Considering the first three they wrote, a total of 519 criteria were grouped by theme for descriptive statistics. Overall, “Animal welfare and care” was the most cited criteria to evaluate a facility (34%, n = 179), followed by “Education” (13%, n = 65), “Staff” (intended as Staff competency, Animal-Staff Interaction and Relationship, and Staff welfare, 10%, n = 54), and “Safety” (of animals and people, 10%, n = 51). “Animal welfare and care” was indicated as the most important criteria to include in the hypothetical rating system by 70% (n = 121) of respondents, followed by “Cleanliness and hygiene” of the facility (5%, n = 8) and “safety” (5%, n = 8). “Animal welfare and care” was indicated also as the second most important criteria to evaluate a facility by 20% (n = 34) of respondents, followed by “education” (16%, n = 27) and “safety” (14%, n = 25). The most cited criteria respondents gave as their third option was “Education” (19%, n = 33), “staff” (14%, n = 25), “Animal welfare and care” (14%, n = 24). The original list of criteria, the categorization process, and the answers’ details are available in S10 Table.

When asked to indicate suggestions to improve their experience (question n.20), respondents equally distributed among the possible answers (Table 5).

**Table 5:** Distribution of answers to question n. 20.

In your opinion, what should be done to improve the experience?	Frequency % (n)
Allowing longer interactions with the animals	10% (18)
Explaining if and how the facility cooperates with conservation programs	16% (28)
Illustrating if and how the facility also works as a rescue centre	13% (23)
Letting animals choose whether to interact or not with us	10% (18)
Offering more information about animal welfare issues	7% (12)
Offering more informative material and in general providing more educational content	5% (8)
Showing videos about the life of the animals in our facility when they are not interacting	24% (41)
Other	14% (25)

The last open-ended question asked the respondents about any additional feedback. 110 out of 173 (64%) respondents reported a comment, which, after a set of 20 questions, denotes a high degree of motivation and engagement.

No other significant results were obtained from the modeling and significance testing of the visitors’ answers.

### 3.4. Staff survey results

A total sample of 14 staff members answered the questionnaires, n = 4 (23.5%) from facility A and n = 10 (58.8%) from facility B.

All the respondents had under their responsibility and cared for semi-captive African elephants involved in AVI. The majority were men (Male = 86%, n = 12; Female = 7%, n = 1; Prefer not to respond = 7%, n = 1), aged between 35 and 54 years old (79% of the respondents, n = 11; between 26–34 years old = 21%, n = 3). The 64% (n = 9) came from Zimbabwe, 36% (n = 5) from South Africa. The majority of respondents have worked in the facility for six to ten years (72%, n = 10), only a few worked in the facility for two to five years (21%, n = 3), and only one worked in the facility for more than ten years (7%, n = 1). Most of the respondents declared to have completed a high school degree (93%, n = 13), while just one pursued further study getting a bachelor’s degree in technology.

Most of the staff sample (64%, n = 9) selected two or more knowledge and know-how sources. The main sources to acquire knowledge and know-how were the education and training programs provided by the facility (86%, n = 12) and colleagues (71%, n = 10). Two respondents (14%) included their family as a source of know-how and knowledge, and two respondents declared that they acquired the knowledge thanks to their previous educational background (14%, n = 2), and eight said they also learned by doing (57%, n = 8).

Questions n. 4–16 explored to which degree the ethical demands of the stakeholder Staff were satisfied. Respondents were asked to express their level of agreement to various statements on a five-point scale. Most of the staff declared that the various demands expressed in the statement were fairly satisfied (mean = 4,18, median = 4, mode = 4). S11 Table indicates the “respect for” principle of the statements, the percentage of respondents choosing each score for each statement, the mean, median, and mode.

When asked to express the main animal welfare problems and/or important topics concerning AVIs (question n. 17) and possible solutions (question n. 18), the respondents indicated zoonotic diseases (four respondents over 14) or stated that there are no animal welfare issues (eight respondents over 14). Two respondents focused on the judgmental attitude of some guests before doing the AVI and on the pressure exerted by animal rights organizations, without explicitly expressing welfare issues. The solutions indicated consisted in the use of preventive measures (e.g., hand sanitizers—four respondents over 14) or recommendations to guests and animal rights organizations to do more informative research to build an educated opinion (two respondents over 14). No other solutions were given. When asked to express management issues concerning AVI (question n. 19), nine respondents out of 14 reported no management issues. The other five respondents indicated as managing challenges the communication between staff members, the animal welfare assessment during the interactions, specific issues related to the management of young untrained calves, guest misinformation, and visitors not listening to and/or following instructions, so behaving inappropriately. To endorse communication between staff and visitors, respondents proposed team meetings and training sessions before the interactions, where effective communication can be practiced and learned, and efficient safety and animal welfare talk before the interaction begins. Moreover, ensuring that the staff is empowered to deal with potentially dangerous situations caused by unpredictable guests and a consistent presence of the manager during the interactions were also recommended. About the challenge of assessing animal welfare, it was remarked the importance of checking the animals before the interactions to ensure they are in good health, behave appropriately, and are not stressed or hurt. In the staff’s opinion, the issues related to the management of calves may be addressed by additional training and by the employment of two dedicated staff members to engage, stimulate and follow the calves.

Staff safety perception was high (question n. 21). Respondents expressed how often they feel unsafe during their daily work with the animals through a five-point scale, ranging from “never “(1) to “always” (5). Nine respondents scored one (never feel unsafe), four respondents scored four, and one respondent reported that it always felt unsafe working with the animals.

The 43% (n = 6) of the respondents declared that in their opinion there were no safety issues (question n.22), one answering that they operate with a high staff to elephant ratio. Another argued that there are no significant risks when safety rules are respected. Non-compliant behaviors of the guests are highlighted as a safety issue from 14% of the respondents (n = 2): guests being where they are not supposed to be and doing what they are not supposed to do (i.e., going to an elephant by themselves, running, screaming shouting, etc.), while 14% of respondents (n = 2) indicated anything that can frighten the elephants (uncontrolled incidents like car crashes, explosions, fires, airplanes) as a source of safety problems. Three respondents declared that meeting a wild animal can be dangerous, or, to use their words, that elephants are “still animals”, with “their own minds, hormones, and emotions, as such, if they are not respected they may injure you”.

To address the listed issues, staff reported what is already done in their facilities, emphasizing some aspects of their safety procedures. Relevant safety procedures include ensuring that people stay in the assigned groups, following staff’s indications, making sure handlers are in front of the animals before the encounter, and not allowing visitors to interact without the staff’s supervision. To improve safety, they also highlight the importance of adequate training of the animals, avoiding performing interaction programs when environmental conditions are adverse (i.e., thunderstorm, heavy rain), and guests respecting the animals. More specifically, it was recommended to desensitize the elephant to as many variables as possible to make the animals more confident and less reactive.

Being part of management strategies to promote the wellbeing of the animals and contribute to conservation and education missions is in the interest of the staff as it promotes their autonomy and their fair treatment. All interviewed staff of facilities A and B declared to have been involved in a staff meeting to promote the wellbeing of the animals included in AVIs in the last year. 50% (n = 7) of the sample declared its engagement also in staff meetings to promote educational activities for visitors, and 43% (n = 6) of the sample declared to have been engaged in meetings focused on animal wellbeing, educational activities, and conservation strategies (Table 6). The last question (question n. 25) asked respondents to write any suggestions on how to improve the AVI. Four respondents over 14 (29%) gave suggestions (reported in S12 Table).

**Table 6:** Distribution of answers to question n. 24.

In the last year have you been engaged in any staff meeting to promote any of the following?	% Respondents (*n*)
…animals’ wellbeing	7% (1)
…animals’ wellbeing AND educational activities	50% (7)
…animals’ well-being AND educational activities AND conservation strategies	43% (6)

### 3.5. Final EM

The ethically relevant demands of the stakeholders (including animals and biodiversity) collected during the workshop were further organized following the frame of the EM. The inputs coming from the open discussions of the workshop, as well as the answers of the surveys, underwent a similar process. A report with the results were sent to the workshop respondents. No additional information, feedback, or review were collected from the participants after sending them the report of this first phase.

The research group carried out multiple phases of brainstorming and revision. Information, concepts, and ideas presented by relevant scientific literature were also evaluated during the analytical process and organized according to the EM framework. This process allowed to define the stakeholders’ interests and value-demand and finalize the Final EM as shown in the synthetic version of the final EM presented in Table 7 and in S13 Table.

**Table 7:** Customized final EM.

	WELL-BEING	AUTONOMY	FAIRNESS
	**Health & welfare**	**Freedom & choice**	**Equity & justice**
	***Maximizing the good*, *minimizing the harm***	***Valuing differences and individual freedom***	***Avoiding discrimination***
**Animals Involved in AVI**	Animal Welfare Animals’ Safety	Behavioural freedom Right to be captive and right to be rewilded	Avoid increasing objectification, animals = sentient beings Equity of treatment Respect their role of “ambassador animals” Animal welfare standards not being influenced by human dissents and conflicts Legal protection
**Owners And Managers**	Satisfactory working conditions Sustainability Well-being of animals, staff and visitors Property interest Having support and the approval of society and Institutions	Managerial freedom Professional development and support Recognition of the peculiar features of each facility	Fair legislation and regulations Equal possibility to communicate Fair assessment of the features of the facility Fair recognition of the actual/potential role of the facility in fulfilling Conservation and/or Education purposes, along with entertainment opportunities
**Staff Involved in AVI**	Safety Satisfactory working conditions Avoid cognitive dissonance *	Professional freedom Professional development Respect for caregivers’ professional ethics Being able to be compliant with the law	Equal opportunities Fair staff recognition Respect for caregiver professional role
**Veterinarians**	Safety Satisfactory working conditions	Professional freedom Possibility to respect professional ethics Being able to be compliant with the law	Being respected as professionals Equitable standards of practice Fair price for their work
**Government representatives**	Development of the Country Personal fulfilment and self-realization Being supported in their work	Being educated and informed Possibility to respect their own institutional role Being provided with resources	Respect of regulations Respect for their institutional role Fair involvement of the different departments
**Biodiversity**	Conservation Mitigating human -animal conflict through education and poverty alleviation of the local rural communities	Autonomy from human intervention Availability of sufficient resources	Equal respect for each component of Nature
**Visitors**	Safety Satisfactory experience Possibility to be emotionally close to animals Avoid cognitive dissonance	Having the opportunity to see wild animals in a controlled environment Possibility to choose Education Informed consent	Affordability Accessibility Equal opportunities
**Animal Rights Groups**	Sustainability of their business Personal fulfilment and self-realization of their members	Freedom to propose their long-term vision on SA tourism industry with regard to AVI Freedom to communicate their ideas regarding AVI Education and access to information, avoiding miseducation	Being recognised as a group of people advocating their own perspective on AVI Equal access to communication

## 4. Discussion

### 4.1 Workshop

Participants attending the workshop had the opportunity to reflect and discuss several topics related to AVIs, including animal welfare and management issues, their interests and value-demands, and those of the other stakeholders. The workshop activities emphasized the cooperative and constructive attitude of the participants, fostering the exchange of ideas and perspectives. This provided stakeholders with an opportunity to step back from their own starting assumptions, relate with other standpoints, and participate in finding a common synthesis.

Reconsidering one’s starting assumption in the light of others’ standpoints is indeed a crucial requirement for the success of a participatory process. The different evaluation of animal welfare and management issues between the 1st and the 2nd round of the preliminary session proves that this result was accomplished. In particular, the most voted clusters at the end of the preliminary session were not the most cited at the start, with some participants choosing to prioritize issues originally proposed by others. In this sense, the workshop successfully created a space for “compromise” between different and often diverging value demands.

The results of the process of identifying and prioritizing animal welfare issues concerning AVIs share common points with WAZA (World Association of Zoos and Aquaria) animal welfare strategy [38] and with the Five Domains welfare model [39, 40]. This consonance gives evidence of the awareness and knowledge of animal welfare of the participants. This shows that the categories involved in the participatory process are key actors to act in the interests of the animals, invest in their welfare, and improve South African tourism facilities. Going back to the animal welfare themes highlighted, it should be noted that: (a) some of them only indirectly affect animal welfare, like communication and animal rights interference; (b) human competency was universally recognized of critical importance; (c) data collected do not provide species-specific insights—not surprising, given the impossibility to define a ‘one size fits all’ welfare strategy [6]; (d) participants showed awareness for zoonosis-related risks, before the COVID-19 pandemic. The fact that participants highlighted themes like communication and animal rights interference as potential causes of concern, not only evidences the analytic attitude of the participants, but also drives attention towards these human dynamics, their potential indirect effects on animal welfare, and the urge to integrate them in the ethical debate.

Human competency was highlighted as a key animal welfare issue and received the highest number of votes. This should raise attention in decision-makers: regulating human competency aspects may contribute to improving animal welfare in South African facilities, and their benefit to the national tourism brand. Moreover, having identified human competency as an important factor for animal welfare, participants *de facto* anticipated some aspects of the 2020 Five Domains Model, which includes the human dimension of animal welfare in its framework [41].

Concerning management, the participants focused on the need for clear legislation and regulation (see themes as legislation, conflicting legislative bodies, conflicting mandates), aside from a series of other issues (i.e. human threats, environmental threats, brand reputation) which may have detrimental effects on the challenge to optimize animal management and care (husbandry and care protocol).

### 4.2. Visitor survey

Visitors participating in the study were highly satisfied with their overall AVI experience, which could be considered an encouraging starting point in terms of respecting their well-being, autonomy, and fair treatment. Interestingly, 58% of the visitor respondents stated that they chose the facility because they were aware of its animal welfare standards. This may sound surprising considering that even experts struggle in assessing animal welfare. Moreover, a gold standard protocol for the welfare assessment of semi-captive elephants (or, in general, of semi-captive wild animals) is lacking, as well as recognized criteria to inform tourists [42]. Even though it would be interesting to understand what respondents intended for animal welfare, this data shows that visitors care for it, and acceptable levels of animal welfare guide visitors in their choices. One possible conservative explanation of this outcome could be that it is mainly the result of a social desirability response bias, a form of motivated misreporting in which people falsely report the socially desirable answer [43]. However, even if based on a social desirability response bias, the outcome remains the same: animal welfare guides visitors in their choice.

This result is in accordance with Miller’s findings that zoo visitors are less likely to support animal facilities when they perceive animal welfare as being poor [44]. It is also supported by another finding of this study. When asked to list criteria for a hypothetic rating system to rate the quality of animal facilities offering AVI, the respondents indicated Animal Welfare or Animal Care as the most important criteria in 70% of the cases.

### 4.3. Staff survey

Handlers are the ones that spend more time with the animals, building unique relationships with them. To perform a detailed ethical analysis of AVIs, it is fundamental to collect their perspective, as they are at the frontline in providing animal care and in ensuring the safety of visitors and animals. Among the insights provided by survey results, it is interesting to discuss their perception of animal welfare and of their wellbeing, autonomy, and fair treatment.

When asked to indicate animal welfare issues, two main trends can be observed: (a) denying the presence of any animal welfare issues; and (b) indicating zoonosis transmission as a major concern. While a focus on zoonosis diseases by handlers seems to reflect common concerns during the COVID 19 pandemic, the fact that most of them did not identify any welfare issue can be explained in different ways. One hypothesis consists in handlers sincerely not perceiving any animal welfare problem. This hypothesis, in turn, would lead the way to another interesting research question: to which extent is this perception linked to high standards of animal welfare offered by the facilities, and to which extent is it instead influenced by the socio-cultural background of handlers? Another hypothesis could be that handlers were reluctant to provide information, given that facilities are currently subject to pressure from activists.

The handlers of facilities A and B seemed satisfied concerning their well-being, autonomy, and fair treatment, indicating that they did not perceive their interests as threatened. This finding is encouraging, also considering the positive outcomes in terms of animal welfare correlated with the satisfaction of caregivers [45, 46]. Handlers seemed particularly satisfied in terms of professional freedom (the majority strongly agreed with the statement “I am able to fully apply my knowledge and skills to my job”). On the other hand, data collected suggest that, according to staff perception, there is room for improvement in terms of economical reward and in terms of feeling appreciated and respected.

### 4.4. EM discussion

The EM developed during this study should provide decision-makers with a framework of the value-demands and ethically relevant aspects involved in AVIs in South Africa to be used as a starting point for the discussion around their regulation.

The Final EM highlights that stakeholders share a common interest: animal welfare. Whether directly or indirectly, respect for animal welfare may provide benefits to all the parties involved. For animals, it is important for obvious reasons tied both to their well-being and autonomy. For veterinarians is important because: a) it is a requirement coming from their professional ethics; b) it evokes positive feelings associated with the accomplishment of duties and vocations, and reduce the risks of compassion fatigue. Similarly, for the staff respecting animal welfare means following professional duties and benefitting from a positive relationship with the animal both on an emotional and on a safety level. By respecting animal welfare, owners and managers may benefit from an increase in the long-term sustainability of the enterprise and brand reputation. Indeed, this study supports the claim that visitors’ perception of animal welfare may be very important from a business perspective [44]. Moreover, data collected during the workshop suggest that certain owners and managers perceive their well-being as linked to those of animals and staff. By advocating animal welfare, government representatives may contribute to their mission of protecting the animals and promoting the South African brand reputation. Likewise, animal rights groups should be interested in animal welfare as well. Finally, respect for animal welfare can also positively affect biodiversity when an AVI is paired with conservation education [21, 25, 47].

As shown by the data from the survey, visitors are also interested in animal welfare, and could especially benefit from having reliable information on the standards adopted by the facility. More specifically, being informed allows them a) to express their freedom to choose whichever facility represents better their expectations; b) to fulfill their right of informed consent; and c) to avoid cognitive dissonance, that is, the unpleasant psychological stress resulting from having an experience with animals and enjoying it while, at the same time, being concerned about their animal welfare.

The methodology followed to develop an EM for AVI allowed to disentangle complex value-issues and helped each stakeholder to put itself in the shoes of every other interest group [21]. The results showed that all the stakeholders involved identified the welfare of the animals involved in AVI as priority. Once animal welfare is recognized as a priority to define the degree of acceptability of AVI practices, decision-makers can evaluate how to incorporate this result in future policies [21]. This result is in line with [31]. The EM developed during this study could therefore helps decision-makers in take decisions and anticipate value conflicts [48] and the focus can be moved on how to assess animal welfare before, during, and after AVIs, and how to communicate animal welfare standards to the tourists.

### 4.5. Strengths, limitations and future developments

During the participatory process, some stakeholder groups, such as animal rights groups and veterinarians, although they had been contacted, were not sufficiently represented, and therefore their requests should be further investigated. Moreover, the staff and visitors interviewed with the questionnaires came almost all from elephant facilities, and, due to the COVID-19 pandemic and associated facility closures, it took more time than planned to collect data and it was not possible to collect surveys from other facilities. Therefore, along with the need to redefine priorities and activities concerning AVIs due to COVID-19 long-term implications, it would be important to collect more data also from facilities offering different AVIs.

An additional point deserving attention is specifically linked to the workshop activities. During the main session, in which participants were asked to advance and discuss their value demands, some stakeholders identified their interests with those of other stakeholders, as can be seen from S6 Table. More specifically, some managers, veterinarians, and staff linked their wellbeing to those of the animals. From one perspective, this could be due to legal or professional reasons. An owner must keep her or his animals healthy to avoid legal repercussions and have more visitors. A caregiver must provide the animals with their needs to respect his or her professional ethics. A veterinarian is compelled by his or her responsibilities to act in the animal’s interests. And so on. Besides these motivations, however, two more hypotheses could explain why some participants identified their interests with those of other stakeholders: (a) it could reflect sincere emphatic feelings; (b) the ethical reasoning task was not fully understood or the stakeholders were not used to this kind of introspective tasks (some stakeholders reported in the feedback form some difficulties on this regard). To improve future similar workshops, these last two hypotheses should be considered. To verify whether and how emphatic feelings towards other stakeholders play a role in the interests of participants, a brief questionnaire could be included at the beginning of the workshop experience. To overcome problems linked to an insufficient comprehension of the task by the participants it would be useful to provide in advance the participants with briefing documents containing information about the EM and the role and goals of ethical analysis. Moreover, during the workshop, it would be useful to allocate more time to dispel doubts and to complete unusual and cognitively demanding tasks.

The results of the participative processes could have been affected by selection bias, so they need to be interpreted cautiously. While selection bias could have affected the results of the workshop and the surveys, this is not negatively affecting the overall results of this study, the final EM of AVI. In fact, both the workshop and the surveys were designed to collect as much inputs as possible to define the final EM presented. All the inputs have been considered for defining the stakeholders’ interests by an inclusive approach.

## 5. Conclusion

The EM showed to be a useful tool to perform a structured ethical analysis on AVIs in South Africa as a first step towards their regulation. In particular, the integrated approach—combining workshop and surveys—adopted in this study assured the direct or indirect engagement of a great part of the affected stakeholders and improved the quality of the representation of their ethical standings. The result is a detailed map of the value demands involved which should facilitate decision-making.

The EM highlights animal welfare as a crucial and transversal issue. In this way, the conclusions of this study fully support the need to develop scientific assessment tools capable to evaluate the welfare of wild animals involved in AVIs considering the peculiar semi-captive and free contact management conditions. Moreover, despite different perspectives on whether and how animals in the “wild-captive continuum” should be managed [42], the EM reflects the overriding importance of compromises and collaboration between the stakeholders to ensure the best possible outcomes for the animals under human responsibility and care.

In addition to the central issue of animal welfare, the study highlights several other relevant issues related to AVIs, like education, biodiversity conservation, sustainability, human competency, facility mission, impact on scientific research, and socio-economic outcomes. In this way, the study shows the importance of an interdisciplinary approach to the issue, and the need to integrate several different criteria to build an official accreditation system dedicated to South African wildlife facilities.

Implementing workshop activities and providing the stakeholders with more opportunities to share their perspectives is of crucial importance to find sustainable solutions and set long-term goals for wildlife tourism evolution in South Africa. As advocated by D’Cruze et al., suitable goals for AVIs should be both biodiversity conservation, education, scientific research, animal welfare, and entertainment [1].

## Supporting information

S1 Table Interim EM. (DOCX)

S2 Table Questions included in the visitor survey, question type, and question purpose. (DOCX)

S3 Table Questions included in the staff survey, question type, and question purpose. (DOCX)

S4 Table Animal welfare issues. (DOCX)

S5 Table Animal management issues. (DOCX)

S6 Table Full list of participants’ notes, from Workshop 3^rd^ round, grouped by Stakeholder and by ethical principle. (DOCX)

S7 Table Demographic information and other independent variables collected from respondents of facilities A, and B. (DOCX)

S8 Table Answers to questions 11–13 accordingly to the need embodied by the respondents. (DOCX)

S9 Table 1. Results of LMMs analysis performed using the level of satisfaction of respondents performing the experience as dependent variable. 2. Results of GLMMs analysis performed using the “need of education” of respondents performing the experience as dependent variable. 3. Results of GLMMs analysis performed using the “biodiversity centric attitude” of respondents performing the experience as dependent variable.
(DOCX)

S10 Table Distribution of categories identified in visitors answers to question n.19 “Imagine an official five star rating system that rates the quality of the facilities in which animal-visitor interactions take place. In your opinion, on which things should the rating be based? Please list the three most important things on which to evaluate the quality of these animal facilities (from most to least important)”.
(DOCX)

S11 Table Answers to questions 4–16, “respect for” principle of the statements, the percentage of respondents choosing each score for each statement, the mean, median, and mode. (DOCX)

S12 Table Suggestions given by respondents in question n. 25. (DOCX)

S13 Table Final detailed EM. (DOCX)

S1 File (DOCX)

S1 Data (XLSX)
