# Supplementary material for: Ethical reasoning and participatory approach towards achieving regulatory processes for animal-visitor interactions (AVIs) in South Africa
Source: PLoS One. 2023 Mar 6;18(3):e0282507. doi: 10.1371/journal.pone.0282507 (PMC9987795; doi:10.1371/journal.pone.0282507)
Supplement: S1 Table — (DOCX) [file pone.0282507.s001.docx]

**S1 Table**. Interim EM

|  | WELLBEING | AUTONOMY | FAIRNESS |
| --- | --- | --- | --- |
|  | Health & welfare | Freedom & choice | Equity & justice |
|  | *Maximizing the good, minimizing the harm* | *Valuing differences and individual freedom* | *Avoiding discrimination* |
| Animals Involved in AVI | Animal Welfare | Behavioural freedom (expression of normal behavioural patterns) | Avoid increasing objectification (animals = sentient beings), equity of treatment (conditions between individuals), entitlement to care |
| Owners And Managers | Property interest, Satisfactory income, Satisfactory working conditions | Managerial freedom.  Freedom to use, freedom to choose the management, freedom to innovate. | Fair regulations (Avoid favouring some individual. Would some facilities be marginalised?). Equal possibility to communicate |
| Staff involved in AVI | Safety, Satisfactory working conditions | Professional freedom (freedom to use their skills and judgement), possibility to behave according to their peculiar relationship with Elephants, possibility to respect professional ethics | Equitable possibilities (es. equitable access to professional updating? fair price for their work? fair distribution of risk? Fair distribution of recognition?) |
| Veterinarians | Safety, Satisfactory working conditions | Professional freedom (freedom to use their skills and judgement), possibility to respect professional ethics (deontological code) | Equitable standards of practice (Fair price for their work) |
| Government representatives | Country development | Possibility to respect own institutional role, free from pressure of single stakeholders (professional ethics) | Respect of regulations  Respect for institutional role |
| Biodiversity | Conservation (Improving scientific knowledge, educational role) | Autonomy from human intervention (Preventing illegal taking of animals from the wild? Avoid direct and indirect contact with wild herds?) | Equal respect for each component of Nature, avoidance of speciesism |
| Visitors | Safety, satisfactory experience, avoiding cognitive dissonance | Informed consent (choice to choose a facility knowing also but not only its "animal welfare standards") and education | Affordability, accessibility to services, education, resources |
| Animal Rights Groups | Sustainability of their business | Freedom to propose their long-term vision on SA tourism industry with regard to AVI | Fair recognition as a group of people advocating their own perspective on AVI  Equal access to communication |
