# Supplementary material for: Ethical reasoning and participatory approach towards achieving regulatory processes for animal-visitor interactions (AVIs) in South Africa
Source: PLoS One. 2023 Mar 6;18(3):e0282507. doi: 10.1371/journal.pone.0282507 (PMC9987795; doi:10.1371/journal.pone.0282507)
Supplement: S2 Table — (DOCX) [file pone.0282507.s002.docx]

| **DEMOGRAPHICAL SECTION** | | | |
| --- | --- | --- | --- |
| **Nr** | **Question** | **Question type** | **Purpose** |
| 1 | Age: ­­_________ | Open-ended | Segment respondents’ sample by age |
| 2 | Nationality :­­­__________ | Dropdown multiple choice | Segment respondents’ sample by nationality |
| 3 | Gender:   - Female - Male - Prefer not to say - Other _______ | Single-answer multiple choice | Segment respondents’ sample by gender |
| 4 | Please choose one of the following options to describe yourself   - Animal lover - Animal expert - Curious tourist - Nature lover - Thrill seeker - Other_______ | Single-answer multiple choice | Segment respondents’ sample by perception of themselves (as visitors) |
| 5 | When did you visit the facility?   - 0-6 months ago - 7-12 months ago - More than 1 year ago - Other_______ | Single-answer multiple choice | Segment respondents’ sample by time passed from visiting the facility |
| 6 | Did you experience an interaction with the animals? (i.e. touching them, walking with them, feeding them, riding etc.)   - Yes - No | Dichotomous | Skip logic branching to direct the respondent or to “PV (Participating Visitors) SECTION” or to “NPV (Non-Participating Visitors) SECTION”. This feature changes what questions a respondent sees next based on how they answer this question.  Visitors who replied “Yes” are directed to “PV (Participating Visitors) SECTION.  Visitors who replied “No” are directed “NPV (Non-Participating Visitors) SECTION”. |

**Table S2.** Questions included in the Visitor Survey, question type, and question purpose

| **PV (PARTICIPATING VISITORS) SECTION** | | | |
| --- | --- | --- | --- |
| **AVIs description** | | | |
| **Nr** | **Question** | **Question type** | **Purpose** |
| **7** | Which animal/s did you interact with, in this facility? (more than one answer possible)   - Elephants - Lions - Dolphins - Cheetahs - Monkeys - Other__________ | Multiple-answer multiple choice | Segment respondents’ sample by animal involved in AVIs experienced in the facility. |
| **8** | What kind of interaction/s did you have with the animals? (more than one answer possible)   - Touching them - Walking with them - Feeding them - Riding - Show - Other_____________ | Multiple-answer multiple choice | Segment respondents’ sample by kind of AVIs experienced in the facility. |

| **PV (Participating Visitors) SECTION** | | | |
| --- | --- | --- | --- |
| **Visitors’ Wellbeing** | | | |
| **Nr** | **Question** | **Question type** | **Purpose** |
| 9 | How happy were you with this experience? (Likert scale 1-5 points; 1= Extremely unhappy; 5= Extremely happy) | Five points Likert scale rating | Investigate how satisfactory the AVI experience was for the respondent. |
| 10 | Did you feel safe during the experience? (Likert scale 1-5 points; 1= Extremely unsafe; 5= Extremely safe) | Five points Likert scale rating | Investigate respondents’ safety perception. |
| 11 | Why did you choose to participate to the interaction/s? Please cross the most important point for you among these three.   - To Entertain my children/ myself (A) - To Educate my children/ myself about animals and nature (B) - To Empathize with animals (C) - Other____ | Single-answer multiple choice | Investigate what respondents prioritize among:  “Need of amusement (A)”, “Need of education (B)”, “Need to be emotionally close to animals (C)” |
| 12 | What were you looking for, having an experience like this? Please cross the most important point for you among these three.   - Having an uncommon experience (A) - Feeling the wonder of being next to such amazing animal/s (C) - Following daily routine of animals, learning about them, their wild cousins and their habitat (B) - Other____ | Single-answer multiple choice | Investigate what respondents prioritize among:  “Need of amusement (A)”, “Need of education (B)”, “Need to be emotionally close to animals (C)” |
| 13 | What impressed you the most? Please cross the most important point for you among these three.   - Having an insight of animal behaviour and discussing about animal welfare challenges (B) - Taking good photos of these incredible animals (A) - Having the opportunity to be physically and emotionally close to the animals, better if without fences inbetween (C) - Other_____ | Single-answer multiple choice | Investigate what respondents prioritize among:  “Need of amusement (A)”, “Need of education (B)”, “Need to be emotionally close to animals (C)” |
| 14 | Among these topics, which one interests you the most?   - Learning about animals (behaviour, anatomy, physiology, reproduction, medical issues, training) - Learning about nature and biodiversity (i.e. Habitat and animals’ role in it, endangered species, sustainable conservation programs) - Learning about the facility history, its current mission and activities - Learning about animals housed in this facility: origin and history, welfare, captivity-related problems. - Other________ | Single-answer multiple choice | Investigate which aspects of education are more important in the mindset of visitors among the proposed ones.  Educational aspects proposed:   - Learning about animals (“animal-centric” mindset) - Learning about biodiversity and conservation (“biodiversity-centric” mindset) - “Understanding Why”: origin, mission and problems… (“ethics-centric” mindset) |

| **PV (Participating Visitors) SECTION** | | | |
| --- | --- | --- | --- |
| **Visitors’ Autonomy** | | | |
| Nr | Question | Question type | Purpose |
| 15 | In your opinion, did you receive enough information about:   \|  \|  \| Yes \| No \| I did not look for information \| \| --- \| --- \| --- \| --- \| --- \| \| 15.1 \| The Facility \|  \|  \|  \| \| 15.2 \| Interaction/s you participated to \|  \|  \|  \| \| 15.3 \| Behavioral rules during the interaction/s \|  \|  \|  \| \| 15.4 \| Welfare of the animal/s you interacted with \|  \|  \|  \| | Trichotomous -matrix | Investigate respondents’ perception of informed consent and accessibility to information. |
| 16 | Where did you get information about the facility and its activities?   - Internet - Travel agency - Hotel - Friends/Family - Directly at the facility, when I reached it - Other________________ | Single-answer multiple choice | Identify the more/less used “information sources”.  Segment respondents’ sample by “information source”.  Investigate accessibility of information. |
| 17 | Why did you choose this specific facility from others that offer similar activities?   - It was the closest one / was on my travel route - It was the cheapest one - Because I was aware of welfare standards in this facility - Because of the possibility to do other interesting activities in the same facility - Because of the number of animals/species housed there - It was the only one I heard about - Other _________________________________________ | Single-answer multiple choice | Investigate how respondents choose an animal facility (selection criteria), to hypnotize what are their needs (informed consent and avoiding cognitive dissonance) |
| **Visitors’ Fairness** | | | |
| 18 | Do you think the price you paid is fair? (Likert scale 1-5 points; 1= Extremely unfair; 5=Extremely fair) | Five points Likert scale rating | Respondents point of view on economical affordability of AVIs |
| 19 | Five stars rating systems are commonly used to rate hotels, restaurants etc. (five stars being the highest quality).  Imagine an official five stars rating system that rates the quality of the facilities in which animal-visitor interactions take place.  In your opinion, on which things should the rating be based?  Please list the three most important things on which to evaluate the quality of these animal facilities (from most to least important).  *List your ideas using the following boxes (A, B, C), ordering them from the most to the least important*  A __________________  B__________________  C __________________ | Open-ended | Identify and rank what respondents perceive as important criteria in a hypothetical rating system dedicated to animal facilities offering AVI.  “Selecting criteria” highlighted by the respondents express their interests in terms of:  - perceived as equal access to information  - perceived important aspects on which to base their decisions (express their freedom to choose)  (WELLBEING+ FAIRNESS + AUTONOMY) |
| **General feedback** | | | |
| 22 | Why did you choose not to experience the interaction with the animals?   - It was not possible to schedule it - I was not interested - I was not willing to pay - I did not know about them - I preferred to relax - I had done it in the past, in another facility - My children were too small - I find hard to trust animals I do not know - I did not want to bother the animals - Other_______ | Single-answer multiple choice | Census of NPV who compiled the questionnaire.  Explore NPV reasons why not participate in the AVIs. Is incidence of any reason significantly higher than incidence of other reasons? |
| 21 | Do you have anything else to add about your experience? Please be 100% honest; we love feedback! | Open-ended | Collect any additional insight on respondents’ point of view, needs, and perception |

| **NPV (NON-PARTICIPATING VISITORS) SECTION** | | | | |
| --- | --- | --- | --- | --- |
| Nr | Question | Question type | Purpose |  |
| 22 | Why did you choose not to experience the interaction with the animals?   - It was not possible to schedule it - I was not interested - I was not willing to pay - I did not know about them - I preferred to relax - I had done it in the past, in another facility - My children were too small - I find hard to trust animals I do not know - I did not want to bother the animals - Other_______ | Single-answer multiple choice | Census of NPV who compiled the questionnaire.  Explore NPV reasons why not participate in the AVIs. Is incidence of any reason significantly higher than incidence of other reasons? |  |
| 23 | Do you have anything else to add about your experience? Please be 100% honest; we love feedback!  __________________________________________________________________________________ | Open-ended | Collect any additional insight on respondents point of view, needs, perception |  |
