# Supplementary material for: Ethical reasoning and participatory approach towards achieving regulatory processes for animal-visitor interactions (AVIs) in South Africa
Source: PLoS One. 2023 Mar 6;18(3):e0282507. doi: 10.1371/journal.pone.0282507 (PMC9987795; doi:10.1371/journal.pone.0282507)
Supplement: S3 Table — (DOCX) [file pone.0282507.s003.docx]

**Table S3.** Questions included in the Staff Survey, question type, and question purpose

| **PRELIMINARY INFORMATION SECTION** | | | |
| --- | --- | --- | --- |
| **Nr** | **Question** | **Question type** | **Purpose** |
| 1 | Please indicate the facility where you are currently working: ____________________ | Open-ended | Segment respondents’ group by facility |
| 2 | Are the animals under your responsibility and care involved in any Animal-Visitor Interaction (AVI)?   - Yes - No   *If “Yes”, continue to “AVIs SECTION”*  *If “No” go to the table “NO-AVIs SECTION”* | Dichotomous | Skip logic branching |
| **AVIs SECTION** | | | |
|  | **ANIMAL-VISITOR INTERACTIONS DESCRIPTION** |  |  |
| **Nr** | **Question** | **Question type** | **Purpose** |
| 3 | Please indicate the animal/s under your responsibility and care which are involved in AVI.  Use the boxes below to specify the animal species followed by the kind of AVI they are involved in (i.e. Elephant -riding)  Note: one box for each species. | Open-ended | Segment respondents’ group by animal species AND/OR kind of AVI. |
|  | **Please indicate the level of agreement regarding the following statements** |  | **(ETHICAL MATRIX questions)** |
| **Nr** | **Question** | **Question type** | **Purpose** |
| 4 | My workplace allows me to balance my work and personal/family life by offering me the support I need when I need it | Five points Likert scale rating (Strongly disagree- Strongly agree) | Respect for WELLBEING- Satisfactory working conditions  (Having a workplace which allows to balance work and personal/family life) |
| 5 | My workplace provides me with the resources, tools and support that I need to do my job to the best of my ability | Five points Likert scale rating (Strongly disagree- Strongly agree) | Respect for AUTONOMY -Professional freedom (Being able to work independently in terms of resources and tools).  Respect for FAIRNESS- Equal opportunities  (Having equal access to funds and resources to develop and grow professionally) |
| 6 | My workplace provides me with satisfactory income | Five points Likert scale rating (Strongly disagree- Strongly agree) | Respect for WELLBEING  Satisfactory working conditions (Having an economically rewarding job) |
| 7 | My workplace environment provides me with satisfactory safety conditions, considering the unique context of my work | Five points Likert scale rating (Strongly disagree- Strongly agree) | Respect for WELLBEING  Safety (Working in a safe and secure environment, also during the interactions) |
| 8 | I am able to fully apply my knowledge and skills to my job | Five points Likert scale rating (Strongly disagree- Strongly agree) | Respect for AUTONOMY  Professional freedom (Freedom to use own skills and judgement, being able to work independently according to knowledge) |
| 9 | My workplace provides me with opportunities for practical training and professional development | Five points Likert scale rating (Strongly disagree- Strongly agree) | Respect for AUTONOMY- Professional development  (Being provided of opportunities for practical training and professional development)  Respect for FAIRNESS - Equal opportunities  (Having equal access to resources to develop and grow professionally) |
| 10 | My workplace allows me to be updated on animal welfare, conservation issues, education, and current legislation. | Five points Likert scale rating (Strongly disagree- Strongly agree) | Respect for AUTONOMY – Professional freedom  Possibility to work independently, being updated on the new research about animal welfare, conservation, education and the current legislation  Respect for FAIRNESS - Equal opportunities  (Having equal access to funds to develop and growth (I.e. professional updating and any resources, tools, support) |
| 11 | I feel that opportunities and recognition go to those who deserve them | Five points Likert scale rating (Strongly disagree- Strongly agree) | Respect for FAIRNESS - Fair staff recognition  (Recognition go to those who deserve it- desert) |
| 12 | My working condition allows me to behave according to the unique relationship I have with the animals I am in care of | Five points Likert scale rating (Strongly disagree- Strongly agree) | Respect for WELLBEING - Avoid cognitive dissonance  (Freedom to behave according to their peculiar relationship with the animals under their care) |
| 13 | My work environment allows me to feel appreciated and respected | Five points Likert scale rating (Strongly disagree- Strongly agree) | Respect for FAIRNESS- Respect for caregiver’s professional role  Being respected as a professional  Respect for WELLBEING  Having a personally rewarding job and being appreciated and respected as a professional |
| 14 | I have the possibility to provide animals with the attention and resources they need | Five points Likert scale rating (Strongly disagree- Strongly agree) | Respect for AUTONOMY  Respect for caregivers’ professional ethics  (Being able to provide animals with the attention and resources they need) |
| 15 | I feel I had an adequate training to work with animals and visitors during the interactions | Five points Likert scale rating (Strongly disagree- Strongly agree) | Respect for AUTONOMY -Professional development  (Feeling to have an adequate training to work both with animals and visitors) |
| 16 | I feel my job is protected by an adequate legislation | Five points Likert scale rating (Strongly disagree- Strongly agree) | Respect for FAIRNESS - Equal opportunities  (Being protected at the working place by a clear and adequate legislation) |
|  | **AVIs ANIMAL WELFARE ISSUES** |  |  |
| **Nr** | **Question** | **Question type** | **Purpose** |
| 17 | In your opinion, what are the main animal welfare problems and/or important topics concerning the Animal-Visitor Interaction?  Think about whatever can reduce/increase the wellbeing of the animal in relation to the Animal-Visitor Interaction.  Please use boxes A, B, C to list the three most important ones (one idea per box).   1. _____________________________________________________________ 2. _____________________________________________________________ 3. _____________________________________________________________ | Open-ended | Investigate animal welfare issues related to AVIs perceived as most relevant AVIs involved staff.  Ranking AVIs animal welfare issues (AVIs involved staff point of view).  This question mirrors Preliminary session of the workshop. |
| 18 | Please list possible solutions/ improvements for the animal welfare problems you indicated­­­­­­­­­­­­­.  Use boxes a, b, c to reply   1. _____________________________________________________________ 2. _____________________________________________________________ 3. _____________________________________________________________ | Open-ended | Investigate staff suggestions to improve AVI animal welfare. |
|  | **AVIs MANAGEMENT ISSUES** |  |  |
| **Nr** | **Question** | **Question type** | **Purpose** |
| 19 | In your opinion, what are the key management problems concerning Animal-Visitor Interaction?  Please indicate the three most important ones using boxes A, B, C (one idea per box).   1. ____________________________________________________________ 2. ____________________________________________________________ 3. ____________________________________________________________ | Open-ended | Investigate management issues related to AVIs perceived as most relevant AVIs involved staff.  Ranking AVIs animal welfare issues (AVIs involved staff point of view).  This question mirrors Preliminary session of the workshop. |
| 20 | Please list possible solutions/ improvements for the management problems you indicated­­­­­­­­­­­­­­­­­­.  Use boxes a, b, c to reply.   1. ___________________________________________________________ 2. ___________________________________________________________ 3. ___________________________________________________________ | Open-ended | Investigate staff suggestions to improve AVI management. |
|  | **SAFETY** |  |  |
| **Nr** | **Question** | **Question type** | **Purpose** |
| 21 | During your daily work with the animals, how often do you feel unsafe? | Five points Likert scale rating  (1= never; 5= always) | Respect for WELLBEING - Safety  Respondents’ safety perception |
| 22 | In your opinion, what are the main dangers concerning Animal-Visitor Interactions?  Think about your safety, visitors’ safety, animals’ safety.  Please use boxes A, B, C to list them (one per box) | Open-ended | Respect for WELLBEING - Safety  Ranking of the dangers concerning AVI, in the respondents’ point of view. Indirect investigation on respondents “safety needs”.  (Moreover, facilitate task of question nr 23) |
| 23 | Do you have suggestions on how to improve safety during Animal-Visitor interactions?  Pease think about your safety, visitors’ safety, animal­­­s’ safety. | Open-ended | Respect for WELLBEING -Safety  Collect the suggestions of the respondents to improve safety of AVIs |
|  | **GENERAL FEEDBACK** |  |  |
| **Nr** | **Question** | **Question type** | **Purpose** |
| 24 | In the last year have you been engaged in any staff meeting to promote any of the following?   - Wellbeing of the animals involved in the interactions - Conservation strategies - Educational activities for the visitors | Multiple-answer multiple choice | Respect for FAIRNESS Equal possibilities to contribute in the mission of the (in terms of welfare, conservation and education)  Respect for AUTONOMY  Possibility to be part to management strategies to promote the wellbeing of the animals and to contribute to conservation and education missions |
| 25 | Do you have suggestions on how to improve the Animal-Visitor Interaction activities? Consider whatever in your opinion is relevant (animal welfare, visitor experience, your work and role in it, management, anything else) | Open-ended | Collecting any suggestion to improve AVI, included respondents’ needs.  Gather additional information to better interpret survey results. |
| **STAFF DEMOGRAPHICALS SECTION** | | | |
| **Nr** | **Question** | **Question type** | **Purpose** |
| 26 | Age   - 14 - 18 years old - 19 - 25 years old - 26 - 34 years old - 35 - 54 years old - 55 - 64 years old - over 64 years old | Single-answer multiple choice | Segment respondents’ group by age |
| 27 | Gender   - Male - Female - Prefer not to say - Other_________ | Single-answer multiple choice | Segment respondents’ group by gender |
| 28 | Nationality ­­­ | Dropdown multiple choice | Segment respondents’ group by nationality |
| 29 | How long have you been working in this facility?   - Less than 1 year - 2 - 5 years - 6 - 10 years - over 10 years | Single-answer multiple choice | Segment respondents’ group by length of working experience in the facility |
| 30 | What is the highest level of education you completed?   - Primary school degree - Middle school degree - High school degree - University degree - PhD/Higher degree - None | Single-answer multiple choice | Segment respondents’ group by educational level |
| 31 | ABOUT YOUR EDUCATION  Please indicate your university degree: _______________________  *(Only if they replied “university degree” or “PhD/Higher degree” to question nr.30)* | Open-ended | Investigate if respondents have specific academic background about animal or nature. Discover what kind of academic background the respondets working with animals has. Compare respondent’s answers with different academic background. |
| 32 | How did you acquire knowledge and know-how about animals?   - I learned by doing - From my colleagues - The facility where I work provides education and training programs - From my family - Thanks to my academic background - Other | Multiple-answer multiple choice | Understand if, among the listed ways to acquire knowledge, any of them have higher incidence in the respondents’ group.  Compare incidence of answer “the facility where I work provides education and training programs” with question nr 5, 9, 10, 15 |
|  | **ADDITIONAL COMMENTS & CONTACTS** |  |  |
| **Nr** | **Question** | **Question type** | **Purpose** |
| 33 | If you wish to add anything to allow better interpretation of your answers or if you wish to add anything at all, please use the space below. | Open-ended | Collect any additional insight on respondents point of view, needs, perception. Collect feedbacks on the survey. |
| 34 | If you wish to receive updates about this study, write to alessia.muzzo@studenti.unipd.it or leave your contact in the free space below. Your contact (optional) :_______ | Open-ended | Respondents’ willingness to receive updates of the study (level of engagement) |
|  | **Please remember to select SUBMIT to validate your answers. Thank you very much!** |  |  |
| **NO AVIs SECTION** | | | |
|  | **ANIMALS** |  |  |
| **Nr** | **Question** | **Question type** | **Purpose** |
| 35 | Please indicate the ANIMALS which are UNDER YOUR RESPONSIBILITY AND CARE:  __________________________________________________________________ | Open-ended | Segment respondents’ group by animal species |
|  | **Please indicate the level of agreement regarding the following statements** |  | **(ETHICAL MATRIX questions)** |
| **Nr** | **Question** | **Question type** | **Purpose** |
| 36 | My workplace allows me to balance my work and personal/family life by offering me the support I need when I need it | Five points Likert scale rating (Strongly disagree- Strongly agree) | Respect for WELLBEING- Satisfactory working conditions  (Having a workplace which allows to balance work and personal/family life) |
| 37 | My workplace provides me with the resources, tools and support that I need to do my job to the best of my ability | Five points Likert scale rating (Strongly disagree- Strongly agree) | Respect for AUTONOMY -Professional freedom (Being able to work independently in terms of resources and tools).  Respect for FAIRNESS- Equal opportunities  (Having equal access to funds and resources to develop and grow professionally) |
| 38 | My workplace provides me with satisfactory income | Five points Likert scale rating (Strongly disagree- Strongly agree) | Respect for WELLBEING  Satisfactory working conditions (Having an economically rewarding job) |
| 39 | My workplace environment provides me with satisfactory safety conditions, considering the unique context of my work | Five points Likert scale rating (Strongly disagree- Strongly agree) | Respect for WELLBEING  Safety (Working in a safe and secure environment, also during the interactions) |
| 40 | I am able to fully apply my knowledge and skills to my job | Five points Likert scale rating (Strongly disagree- Strongly agree) | Respect for AUTONOMY  Professional freedom (Freedom to use own skills and judgement, being able to work independently according to knowledge) |
| 41 | My workplace provides me with opportunities for practical training and professional development | Five points Likert scale rating (Strongly disagree- Strongly agree) | Respect for AUTONOMY- Professional development  (Being provided of opportunities for practical training and professional development)  Respect for FAIRNESS - Equal opportunities  (Having equal access to resources to develop and grow professionally) |
| 42 | My workplace allows me to be updated on animal welfare, conservation issues, education, and current legislation. | Five points Likert scale rating (Strongly disagree- Strongly agree) | Respect for AUTONOMY – Professional freedom  Possibility to work independently, being updated on the new research about animal welfare, conservation, education and the current legislation  Respect for FAIRNESS - Equal opportunities  (Having equal access to funds to develop and growth (I.e. professional updating and any resources, tools, support) |
| 43 | I feel that opportunities and recognition go to those who deserve them | Five points Likert scale rating (Strongly disagree- Strongly agree) | Respect for FAIRNESS - Fair staff recognition  (Recognition go to those who deserve it- desert) |
| 44 | My working condition allows me to behave according to the unique relationship I have with the animals I am in care of | Five points Likert scale rating (Strongly disagree- Strongly agree) | Respect for WELLBEING - Avoid cognitive dissonance  (Freedom to behave according to their peculiar relationship with the animals under their care) |
| 45 | My work environment allows me to feel appreciated and respected | Five points Likert scale rating (Strongly disagree- Strongly agree) | Respect for FAIRNESS- Respect for caregiver’s professional role  Being respected as a professional  Respect for WELLBEING  Having a personally rewarding job and being appreciated and respected as a professional |
| 46 | I have the possibility to provide animals with the attention and resources they need | Five points Likert scale rating (Strongly disagree- Strongly agree) | Respect for AUTONOMY  Respect for caregivers’ professional ethics  (Being able to provide animals with the attention and resources they need) |
| 47 | I feel I had an adequate training to work with animals and visitors during the interactions | Five points Likert scale rating (Strongly disagree- Strongly agree) | Respect for AUTONOMY -Professional development  (Feeling to have an adequate training to work both with animals and visitors) |
| 48 | I feel my job is protected by an adequate legislation | Five points Likert scale rating (Strongly disagree- Strongly agree) | Respect for FAIRNESS - Equal opportunities  (Being protected at the working place by a clear and adequate legislation) |
|  | **GENERAL FEEDBACK** |  |  |
| **Nr** | **Question** | **Question type** | **Purpose** |
| 49 | In the last year have you been engaged in any staff meeting to promote any of the following?   - Wellbeing of the animals involved in the interactions - Conservation strategies - Educational activities for the visitors | Multiple-answer multiple choice | Respect for FAIRNESS Equal possibilities to contribute in the mission of the (in terms of welfare, conservation and education)  Respect for AUTONOMY  Possibility to be part to management strategies to promote the wellbeing of the animals and to contribute to conservation and education missions |
| 50 | Do you have suggestions on how to improve the Animal-Visitor Interaction activities? Consider whatever in your opinion is relevant (animal welfare, visitor experience, your work and role in it, management, anything else) | Open-ended | Collecting any suggestion to improve AVI, included respondents’ needs.  Gather additional information to better interpret survey results. |
| **STAFF DEMOGRAPHICALS SECTION** | | | |
| **Nr** | **Question** | **Question type** | **Purpose** |
| 51 | Age   - 14 - 18 years old - 19 - 25 years old - 26 - 34 years old - 35 - 54 years old - 55 - 64 years old - over 64 years old | Single-answer multiple choice | Segment respondents’ group by age |
| 52 | Gender   - Male - Female - Prefer not to say - Other_________ | Single-answer multiple choice | Segment respondents’ group by gender |
| 53 | Nationality ­­­ | Dropdown multiple choice | Segment respondents’ group by nationality |
| 54 | How long have you been working in this facility?   - Less than 1 year - 2 - 5 years - 6 - 10 years - over 10 years | Single-answer multiple choice | Segment respondents’ group by length of working experience in the facility |
| 55 | What is the highest level of education you completed?   - Primary school degree - Middle school degree - High school degree - University degree - PhD/Higher degree - None | Single-answer multiple choice | Segment respondents’ group by educational level |
| 56 | ABOUT YOUR EDUCATION  Please indicate your university degree: _______________________  *(Only if they replied “university degree” or “PhD/Higher degree” to question nr.30)* | Open-ended | Investigate if respondents have specific academic background about animal or nature. Discover what kind of academic background the respondets working with animals has. Compare respondent’s answers with different academic background. |
| 57 | How did you acquire knowledge and know-how about animals?   - I learned by doing - From my colleagues - The facility where I work provides education and training programs - From my family - Thanks to my academic background - Other | Multiple-answer multiple choice | Understand if, among the listed ways to acquire knowledge, any of them have higher incidence in the respondents’ group.  Compare incidence of answer “the facility where I work provides education and training programs” with question nr 5, 9, 10, 15 |
|  | **ADDITIONAL COMMENTS & CONTACTS** |  |  |
| **Nr** | **Question** | **Question type** | **Purpose** |
| 58 | If you wish to add anything to allow better interpretation of your answers or if you wish to add anything at all, please use the space below. | Open-ended | Collect any additional insight on respondents point of view, needs, perception. Collect feedbacks on the survey. |
| 59 | If you wish to receive updates about this study, write to alessia.muzzo@studenti.unipd.it or leave your contact in the free space below. Your contact (optional) :_______ | Open-ended | Respondents’ willingness to receive updates of the study (level of engagement) |
|  | **Please remember to select SUBMIT to validate your answers. Thank you very much!** |  |  |
