# Supplementary material for: Ethical reasoning and participatory approach towards achieving regulatory processes for animal-visitor interactions (AVIs) in South Africa
Source: PLoS One. 2023 Mar 6;18(3):e0282507. doi: 10.1371/journal.pone.0282507 (PMC9987795; doi:10.1371/journal.pone.0282507)
Supplement: S4 Table — (DOCX) [file pone.0282507.s004.docx]

S4 Table. Animal Welfare issues

| Animal Welfare Issues | Animal Welfare Clusters | Stakeholders who identified the issues | Stakeholders who voted for the cluster | Number of votes for cluster |
| --- | --- | --- | --- | --- |
| Activists limiting interactions which we use as opportunity to enrich animals lives | Animal rights interference | Government Representatives; Owners & Managers | Government Representatives; Owners & Managers; researchers | 4 |
| Animal rights interference |  |  |  |  |
| No support from public- where are the animals going to go? (part 1) |  |  |  |  |
| Priority of protection is given to Elephants then Human (part 1) |  |  |  |  |
| Behavioral checks (hormones-glucocorticoids can be both positive and negative and no one knows which it is) (part 1) | Assessment (animal) | Owners & Managers; Researchers; Veterinarians | Owners & Managers; Researchers | 3 |
| Geriatric care and quality of life evaluation (part 1) |  |  |  |  |
| Integrated assessment (individual + environment+ behavior +….) |  |  |  |  |
| Mortality in a captive setting - what is seen as an acceptable level of care? i.e. exhibit with 1000 fish - is it acceptable to have one mortality a day? How you define and evaluate good level of care? (part 1) |  |  |  |  |
| Objective measure of "stress", "health", "welfare" |  |  |  |  |
| Reptiles. How do we provide them choice? |  |  |  |  |
| Limited funds could lead to limited resources (e.g. food/medicines/ vet services) | Best practice | Government Representatives; Researchers | Owners & Managers; Researchers; Veterinarians | 5 |
| Management of animals in the absence of appropriate welfare standards (part 1) |  |  |  |  |
| Expectation of guest in regarding interacting with wild animals | Communication | Owners & Managers; Researchers | Government Representatives; Owners & Managers | 2 |
| Ignorance of "Bunny huggers" (part 1) |  |  |  |  |
| Lack of understanding of welfare issues by public. i.e. they assume |  |  |  |  |
| No support from public- where are the animals going to go? (part 2) |  |  |  |  |
| Management of animals in the absence of appropriate welfare standards (part 2) | Compliance | Government Representatives; Owners & Managers | Handlers/Keepers/Staff; Owners and managers; Veterinarians | 5 |
| Uncontrolled legislation |  |  |  |  |
| Environmental enrichment | Enrichment | Researchers |  | 0 |
| Limitations in cognitive experiences for (captive) animals (part 1) |  |  |  |  |
| Limitations to social structure of animal groups (captive) (part 1) |  |  |  |  |
| Limited interactions with other animals (part 1) |  |  |  |  |
| Five freedoms | Five domains | Handlers/Keepers/Staff; Owners and managers | Owners and managers | 1 |
| Health: Mind (stress, enrichment), Body (Fit, Healthy, Fed & Watered), Spirit (Happy, other animal interaction, preff a mate) (part 1) |  |  |  |  |
| How to ensure that all Five Freedoms regarding the captive animals are met. |  |  |  |  |
| Animal fatigue | Health | Owners and Managers; Researchers; Veterinarians | Owners and Managers; Researchers; | 5 |
| Animal injury |  |  |  |  |
| Behavioral checks (hormones-glucocorticoids can be both positive and negative and no one knows which it is) (part 2) |  |  |  |  |
| Feeding - proper nutrition (part 1) |  |  |  |  |
| Geriatric care and quality of life evaluation (part 2) |  |  |  |  |
| Health: Mind (stress, enrichment), Body (Fit, Healthy, Fed & Watered), Spirit (Happy, other animal interaction, preff mate) (part 2) |  |  |  |  |
| Mortality in a captive setting - what is seen as an acceptable level of care? i.e. exhibit with 1000 fish - is it acceptable to have one mortality a day? How you define and evaluate good level of care? (part 2) |  |  |  |  |
| Over feeding |  |  |  |  |
| Regular vet assessments |  |  |  |  |
| Routine health assessment |  |  |  |  |
| Veterinary checks |  |  |  |  |
| Lay people in the medical field. Medical issues: opinion that an animal is sick -i.e. it is suffering and this should not happen-lets just euthanize it. Lay people are too sensitive to see medical being done | Human competency | Owners and Managers; Veterinarians | Government Representatives; Handlers/Keepers/Staff; Owners and Managers; Researchers | 8 |
| Training staff/handlers on welfare and husbandry of animals |  |  |  |  |
| Adequate shelter/ enclosure (part 1) | Implementing husbandry | Handlers/Keepers/Staff; Owners and Managers; Researchers | Handlers/Keepers/Staff; Owners and Managers; | 3 |
| Animal Refuges opportunities (part 1) |  |  |  |  |
| Feeding - proper nutrition (part 2) |  |  |  |  |
| Limitations in cognitive experiences for (captive) animals (part 2) |  |  |  |  |
| Limitations to social structure of animal groups (captive) (part 2) |  |  |  |  |
| Limited interactions with other animals (part 2) |  |  |  |  |
| Littering |  |  |  |  |
| Provision of browse |  |  |  |  |
| Provision of browse for browsers |  |  |  |  |
| Carrying capacity vs population | Population control (management) | Handlers/Keepers/Staff; Researchers | Government Representatives; Researchers | 2 |
| Crowding / density |  |  |  |  |
| Population control --> culling |  |  |  |  |
| Absence of regulatory provisions to prohibit the manner in which activities may be carried out--> absence of acceptable margins | Regulating "rules" | Government Representatives | Government Representatives; Handlers/Keepers/Staff; Owners and Managers | 3 |
| Full participation of the department that deals with welfare |  |  |  |  |
| Animal Refuges opportunities (part 2) | Regulating interactions | Owners and Managers; Researchers | Owners and Managers | 2 |
| Disturbance of resting phases of animals (by human presence) |  |  |  |  |
| Elephant "rides" (part 1) |  |  |  |  |
| Length of interaction |  |  |  |  |
| Number of interactions |  |  |  |  |
| Number of participants at one time |  |  |  |  |
| Public feeding animals |  |  |  |  |
| Ignorance of "Bunny huggers" (part 2) | Safety (Animal, Human) | Governments Representatives; Owners and Managers; Researchers | Handlers/Keepers/Staff; Owners and Managers | 4 |
| Poaching |  |  |  |  |
| Priority of protection is given to Elephants then Human (part 2) |  |  |  |  |
| Safety of animals |  |  |  |  |
| Unpredictable guests a threat to animals |  |  |  |  |
| Adequate shelter/ enclosure (part 2) | Space | Owners and Managers; Researchers | Veterinarians | 1 |
| Animal to have sufficient space to move around |  |  |  |  |
| Providing a natural environment for our animals / space |  |  |  |  |
| Space |  |  |  |  |
| Animal Training | Training (animal) | Handlers/Keepers/Staff; Owners and Managers; Researchers | Handlers/Keepers/Staff; Owners and Managers | 3 |
| Elephant "rides" (part 2) |  |  |  |  |
| Training |  |  |  |  |
| Disease outbreaks | Zoonosis and diseases | Owners and Managers; Researchers | Researchers | 2 |
| Disease transmission (human-animal both ways) |  |  |  |  |
| Spread of disease |  |  |  |  |
| Zoonosis; diseases from people to animals |  |  |  |  |
| Zoonotic diseases |  |  |  |  |
