# Supplementary material for: Ethical reasoning and participatory approach towards achieving regulatory processes for animal-visitor interactions (AVIs) in South Africa
Source: PLoS One. 2023 Mar 6;18(3):e0282507. doi: 10.1371/journal.pone.0282507 (PMC9987795; doi:10.1371/journal.pone.0282507)
Supplement: S5 Table — (DOCX) [file pone.0282507.s005.docx]

**S5 Table.** Animal Management issues

| Management issues | Management cluster | Stakeholders who identified the issues | Stakeholders who voted for the cluster | Number of votes for cluster |
| --- | --- | --- | --- | --- |
| Managing the brand / reputation and protecting against lobbyist/activists | Brand Reputation | Owners & Managers; | Owners & Managers; | 2 |
| Negative Influencers |  |  |  |  |
| Negative publicity from media |  |  |  |  |
| Pressure from animal groups (part 2) |  |  |  |  |
| Public perception (part 3) |  |  |  |  |
| Declining visitor numbers (part 2) |  |  |  |  |
| Sat , SATSA and other organizations are against interactions - damaging our business and brand (part 1) |  |  |  |  |
| Animal welfare, ensuring guests = ensuring food, care, good facilities etc. (part 2) | Communication | Owners & Managers; Handlers/Keepers/Staff; | Owners & Managers; Researchers | 4 |
| Managing the expectations of guests re interactions and how to safely interact with animals (part 1) |  |  |  |  |
| Public perception (part 1) |  |  |  |  |
| To get the right conservation message across for biodiversity not species (part 1) |  |  |  |  |
| What is the public perception regarding captive vs wild elephant |  |  |  |  |
| Visitor training and compliance (part 2) |  |  |  |  |
| Guest expectation. They expect more or different from what we offer (part 1) |  |  |  |  |
| Challenges in Legislation | Conflicting legislatory bodies | Owners & Managers; | Owners & Managers; Researchers; Handlers/Keepers/Staff; Government Representatives | 5 |
| Regulations |  |  |  |  |
| Change in political mandate - influences budget allocation and priorities | Conflicting mandates | Government Representatives; Owners & Managers; Veterinarians | Researchers; Government Representatives | 2 |
| Conflictory mandators between Agric and Environmental Affairs on welfare management |  |  |  |  |
| Regulations by unregistered bodies |  |  |  |  |
| Everyone has an opinion on welfare - equating the opinions of a lay person with that of someone that has actually worked on the issue for some time I.e. define stakeholder importance |  |  |  |  |
| Sat , SATSA and other organizations are against interactions - damaging our business and brand (part 2) |  |  |  |  |
| Assumptions. Is an animal in the wild - unmanaged state in a better welfare state than an animal under the right human care ? (mgt). Dog on streets is free to do all it wants, yet NSPCA feel it is better in a kennel | Conservation education | Owners & Managers; Researchers | Owners & Managers; Researchers | 4 |
| Communicating what a happy animal is. I.e. Elephant temporal gland; public see issue, Elephant handler sees elephant is happy |  |  |  |  |
| Interaction = connection. Enables education aspect as well as research opportunities |  |  |  |  |
| Public perception (part 2) |  |  |  |  |
| Purpose of interaction (2) |  |  |  |  |
| Sensitizing to ethics /welfare |  |  |  |  |
| To get the right conservation message across for biodiversity not species (part 2) |  |  |  |  |
| Uneducated public |  |  |  |  |
| Guest expectation. They expect more or different from what we offer (part 2) |  |  |  |  |
| Education of people and staff (part 1) |  |  |  |  |
| Education of managers / public (part 1) |  |  |  |  |
| Environmental factors (Drought, lack of rainfall, excessive heat) | Environmental threats | Owners & Managers; Researchers; |  | 0 |
| Unplanned veldfires |  |  |  |  |
| Environmental factors (Drought, lack of rainfall, excessive heat) |  |  |  |  |
| Business interruptions water cut and power cut | Governance | Owners & Managers; Government Representatives; Veterinarians | Owners & Managers; Researchers; Government Representatives; Veterinarians | 6 |
| Staff opinions/ emotions that prevent/ delay actions taken - medical action needs to be made quickly but every staff has an opinion and meeting/discussions delay treatment (part 2) |  |  |  |  |
| People keeping animals not compliant with the provision of the legislation (part 1) |  |  |  |  |
| Management of animals in contravention of legislation (part 2) |  |  |  |  |
| Activities of displaced people (recycling, wood collection, collection medicinal plant, poaching) | Human threats | Owners & Managers; Veterinarians | Owners & Managers; Researchers; Handlers/Keepers/Staff | 3 |
| Ego and personal agendas are more important than the animals wellbeing. Opinions of people- and their emotions are placed before the welfare of animals (part 2) |  |  |  |  |
| Lack of space for expansion and development (competition for space with other community needs ex Housing) (part 2) |  |  |  |  |
| Pressure from animal groups (part 1) |  |  |  |  |
| Animal welfare, ensuring guests = ensuring food, care, good facilities etc. (part 1) | Husbandry and care protocol | Owners & Managers; Researchers; Government Representatives; | Owners & Managers; Researchers; Handlers/Keepers/Staff; Veterinarians | 7 |
| Enclosed areas (fences) (part 2) |  |  |  |  |
| Hormone control (impact on animals temperament, no babies= problem for elephant) |  |  |  |  |
| Husbandry and welfare of animals training |  |  |  |  |
| Lack of space for expansion and development (competition for space with other community needs ex Housing) (part 1) |  |  |  |  |
| Notion of managing animals as if they are same as domestic stock (part 1) |  |  |  |  |
| Understanding + measuring effect on animals |  |  |  |  |
| Zoonosis - disease from animal to people (part 1) |  |  |  |  |
| Reference values for decisions (quarantine /cull) (part 1) |  |  |  |  |
| Limitations at welfare assessment tools for stress measures |  |  |  |  |
| Limited space for animals (part 2) |  |  |  |  |
| Artificial water sources |  |  |  |  |
| Ego and personal agendas are more important than the animals wellbeing. Opinions of people- and their emotions are placed before the welfare of animals (part 1) | Internal codes of conduct | Owners & Managers; Researchers; Government Representatives; Veterinarians | Veterinarians | 1 |
| For elephants specifically: Matching temperament with allowed interaction |  |  |  |  |
| Purpose of interaction (1) |  |  |  |  |
| Staff opinions/ emotions that prevent/ delay actions taken - medical action needs to be made quickly but every staff has an opinion and meeting/discussions delay treatment (part 1) |  |  |  |  |
| Visitor training and compliance (part 1) |  |  |  |  |
| Limited interaction time |  |  |  |  |
| Management of animals in absence of clear objectives. I.e., without a management plan (part 1) |  |  |  |  |
| Legislation= Prescriptive which can be detrimental to animal | Legislation | Owners & Managers; Researchers; Government Representatives; | Owners & Managers; Researchers; Handlers/Keepers/Staff; | 5 |
| No proper regulation from gov. No support from gov. |  |  |  |  |
| Reference values for decisions (quarantine /cull) (part 2) |  |  |  |  |
| People keeping animals not compliant with the provision of the legislation (part 2) |  |  |  |  |
| Liability to business (legally) |  |  |  |  |
| Management of animals in contravention of legislation (part 1) |  |  |  |  |
| Enclosed areas (fences) (part 1) | Safety (Animal and humans) | Owners & Managers; Researchers | Owners & Managers | 2 |
| Managing the expectations of guests re interactions and how to safely interact with animals (part 2) |  |  |  |  |
| Safety of both animals and staff and public |  |  |  |  |
| Safety of guest, dealing with animals who are not always predictable |  |  |  |  |
| Working with animals. Humans: different thinking, backgrounds, predictability; Animals: own brain, instincts and hormones |  |  |  |  |
| Zoonosis - disease from animal to people (part 2) |  |  |  |  |
| Budget | Sustainability | Owners & Managers; Handlers/Keepers/Staff; Government Representatives | Owners & Managers; Researchers; Handlers/Keepers/Staff; Government Representatives | 6 |
| Financially: looking after so many animals |  |  |  |  |
| Insufficient budget |  |  |  |  |
| Declining visitor numbers (part 1) |  |  |  |  |
| Management of animals in absence of clear objectives. I.e., without a management plan (part 2) |  |  |  |  |
| Caretakers not trained in the exact field for interacting with animals | Training people | Owners & Managers; Researchers; Handlers/Keepers/Staff; Government Representatives | Owners & Managers; Researchers; Government Representatives | 4 |
| Identifying skilled personnel with experience on working with guest and animals |  |  |  |  |
| No experienced personnel dealing with new or updated regulations in regard to keeping wild animals |  |  |  |  |
| Notion of managing animals as if they are same as domestic stock (part 2) |  |  |  |  |
| Number of trained staff |  |  |  |  |
| Personnel |  |  |  |  |
| Staff perception |  |  |  |  |
| Staff skills. Ensuring they have skills to make experience as meaningful as possible |  |  |  |  |
| Staff training (understanding animals, not just a job, same level of education/qualification, understanding of concepts) |  |  |  |  |
| Training of staff (Staff, Public + Animals) |  |  |  |  |
| Well trained keepers |  |  |  |  |
| Education of people and staff (part 2) |  |  |  |  |
| Education of managers / public (part 2) |  |  |  |  |
| Operators/ agents not using products? | Excluded (others) |  |  | 0 |
| Value of interaction |  |  |  |  |
| Record helping opinions on behaviour, time consuming and can be over analysed, level of education of record keeper, costly |  |  |  |  |
