# Supplementary material for: Ethical reasoning and participatory approach towards achieving regulatory processes for animal-visitor interactions (AVIs) in South Africa
Source: PLoS One. 2023 Mar 6;18(3):e0282507. doi: 10.1371/journal.pone.0282507 (PMC9987795; doi:10.1371/journal.pone.0282507)
Supplement: S6 Table — (DOCX) [file pone.0282507.s006.docx]

**Table S6.** Full list of participants’ notes, from Workshop 3^rd^ Round, grouped by Stakeholder and by Ethical Principle

| STAKEHOLDER | WELLBEING | AUTONOMY | FAIRNESS |
| --- | --- | --- | --- |
| Government  Representatives | - Best practices; ensuring compliance of the legislation - Compliance; ensuring that the animals will be protected - Governance | - Animal rights interference; valuing animal rights views - Legislation; agreeing on different legislation | - Legislation; fair legislation , not punishing the species or people |
| Handlers/  Keepers/  Staff | - Health; to be vaccinated against zoonotic diseases; work in the conductive environment - Safety; wear protective clothes , avoid direct contact with animals | - Best practices; to be the best in the field (world class) - Communication; open communication channels between management and keepers - Compliance; protocols to be implemented and record keeping - Husbandry and care protocol; best husbandry practices, feed animals according to their needs etc. | - Animals have got rights to live in captivity - Governance; comply with legislation - Respected opinion of member of public |
| Veterinarians | - Best practice; I will need to have a access to resources that ensure that treatment step taken to treat an animal will result in the best possible outcome - Governance; I will need clarity of the legislation involved in animal interaction and what is expected of the Vet | - Best practice; I would like to have the freedom to implement treatment / diagnostics in such a way that will ensure the best possible diagnostics and treatment outcome - Governance (permits and regulation); I will need to be able to verify and challenge regulations to legally compel me to act in a certain way | - Best practice; I feel that it is only fair that once I have consulted the best specialist and researched the best treatment options, that these are actually implemented |
| Owners &  Managers | - Best practices for staff and animal safety - Communication; effective accountable communication process that supports progress - Competent and dedicated staff - Constant communication with relevant stakeholders - Create interactions that serve to enrich lives of animals. I would need industry and company support (best practice) - Handlers to have a good training plus examples - Need business sustainability - Need competent staff - Safety (Animal and Human) - Safety; great training of handlers so they are competent in their job in training and care - Safety; well cared for and well fed; hormones and trusting animals - Secure / sustainable business - Sustainability of the industry - Time - Understanding the animals | - Best practice - Best practice; provide me support to ensure that staff enables animals with choice + control in daily interactions - Business Continuity - Communication; clear accountable process that operates at all levels - Husbandry and care protocol; I would need a self regulating body to speak to about issues who can understand why I may need to manage in a way I do - Legislation - Legislation; legislation that understands that managing and caring for animals is not a black and white issue - Need to by recognized as an expert facility - Operate ethically according to legislation and communicate with society - Operate the business without interference from stakeholders / directors - Simple legislation compliance - Simple regulation - Sustainability - To operate without any unfair / false accusation | - Animal rights; understanding of key points for welfare of my animals - Best practice; the opportunity to manage my team and animals in a way that I know benefits them - Best practices - Clear communication between representation bodies - Communication - a body that is able to give both points of view on a subject and not an opinionated statement - Communication; disciplined positively regular processes to support good communication - Compliance - Conflicting legislation bodies; I would need… bodies + stakeholders to discuss issues once a year - Educated staff - I need a working environment for me, my staff and animals, that operates ethically, compliant and provides best practices for all - I would require support to measure success of process to ensure that interactions are enriching - Need competent auditors - Sustainability; support for what we do and understanding of what we aim to do |
