# Supplementary material for: Ethical reasoning and participatory approach towards achieving regulatory processes for animal-visitor interactions (AVIs) in South Africa
Source: PLoS One. 2023 Mar 6;18(3):e0282507. doi: 10.1371/journal.pone.0282507 (PMC9987795; doi:10.1371/journal.pone.0282507)
Supplement: S7 Table — (DOCX) [file pone.0282507.s007.docx]

Table S7. Demographic information and other independent variables collected from respondents of facilities A, and B

| **Demographic** | **Category** | **Frequency (%)** | ***n*** |
| --- | --- | --- | --- |
| **Age (range in years)** | 14-18 | 1 | 1 |
|  | 19-25 | 6 | 10 |
|  | 26-34 | 13 | 23 |
|  | 35-54 | 46 | 81 |
|  | 55-64 | 25 | 45 |
|  | over 64 | 10 | 17 |
| **Nationality** | Africa | 68 | 120 |
|  | Australia | 1 | 2 |
|  | Europe | 17 | 30 |
|  | North America | 12 | 21 |
|  | South America | 2 | 4 |
| **Gender** | Female | 63 | 112 |
|  | Male | 37 | 65 |
| **Self-description** | Animal expert | 6 | 11 |
|  | Animal lover | 50 | 89 |
|  | Curious tourist | 3 | 5 |
|  | Nature lover | 36 | 64 |
|  | Other | 3 | 6 |
|  | Thrill seeker | 1 | 2 |
| **Last visit** | More than 1 year | 37 | 66 |
|  | 7-12 months | 25 | 45 |
|  | Less than 6 months | 37 | 66 |
