# Supplementary material for: Ethical reasoning and participatory approach towards achieving regulatory processes for animal-visitor interactions (AVIs) in South Africa
Source: PLoS One. 2023 Mar 6;18(3):e0282507. doi: 10.1371/journal.pone.0282507 (PMC9987795; doi:10.1371/journal.pone.0282507)
Supplement: S8 Table — (DOCX) [file pone.0282507.s008.docx]

**Table S8.** Answers to questions 11-13 accordingly to the need embodied by the respondents

|  |  | **Need of Amusement**  **% (*n*)** | **Need of education**  **% (*n*)** | **Need to be emotionally close to animals**  **% (*n*)** |
| --- | --- | --- | --- | --- |
| **Question 11** | Why did you choose to participate? | 8% (13) | 76% (132) | 16% (28) |
| **Question 12** | What were you looking for? | 4% (7) | 35% (60) | 61% (106) |
| **Question 13** | What impressed you the most? | 2% (4) | 46% (79) | 52% (90) |
