# Supplementary material for: Ethical reasoning and participatory approach towards achieving regulatory processes for animal-visitor interactions (AVIs) in South Africa
Source: PLoS One. 2023 Mar 6;18(3):e0282507. doi: 10.1371/journal.pone.0282507 (PMC9987795; doi:10.1371/journal.pone.0282507)
Supplement: S9 Table — 1. Results of LMMs analysis performed using the level of satisfaction of respondents performing the experience as dependent variable. 2. Results of GLMMs analysis performed using the “need of education” of respondents performing the experience as dependent variable. 3. Results of GLMMs analysis performed using the “biodiversity centric attitude” of respondents performing the experience as dependent variable. (DOCX) [file pone.0282507.s009.docx]

Table S9.1. Results of LMMs analysis performed using the level of satisfaction of respondents performing the experience as dependent variable.

|  | Estimate | NumDF | DenDF | F value | P |
| --- | --- | --- | --- | --- | --- |
| Age | - | 1 | 157.28 | 0.025 | 0.876 |
| Gender | + | 1 | 156.81 | 0.268 | 0.606 |
| Timpe | - | 1 | 155.03 | 0.012 | 0.914 |
| Animal experts | + | 1 | 157.53 | 0.063 | 0.803 |
| Continent of belonging |  | 4 | 128.52 | 0.813 | 0.519 |
| Need of amusement | - | 1 | 156.34 | 7.519 | **0.007** |
| Need of education | + | 1 | 154.41 | 0.320 | 0.572 |
| Emotionally close | + | 1 | 156.28 | 0.598 | 0.440 |
| Conservation driven | - | 1 | 156.75 | 3.440 | 0.066 |
| Education driven | + | 1 | 157.15 | 0.038 | 0.847 |
| Animal Driven | - | 1 | 155.87 | 0.125 | 0.725 |

Table S9.2. Results of GLMMs analysis performed using the “need of education” of respondents performing the experience as dependent variable

|  | Estimate | c^2^ | Df | P |
| --- | --- | --- | --- | --- |
| X1_Age | + | 2.471 | 1.00 | 0.116 |
| X3_Gender | - | 0.088 | 1.00 | 0.767 |
| X5_time | - | 0.227 | 1.00 | 0.634 |
| Animal experts | + | 5.441 | 1.00 | **0.019** |
| Continent of belonging |  | 2.413 | 4.00 | 0.660 |

Table S9.3. Results of GLMMs analysis performed using the “biodiversity centric attitude” of respondents performing the experience as dependent variable.

|  | Estimate | c^2^ | Df | P |
| --- | --- | --- | --- | --- |
| X1_Age | + | 5.770 | 1 | **0.016** |
| X3_Gender | + | 0.294 | 1 | 0.587 |
| X5_time | - | 0.060 | 1 | 0.806 |
| perception_acc | + | 0.009 | 1 | 0.927 |
| Continent |  | 3.956 | 4 | 0.412 |
