# Supplementary material for: Ethical reasoning and participatory approach towards achieving regulatory processes for animal-visitor interactions (AVIs) in South Africa
Source: PLoS One. 2023 Mar 6;18(3):e0282507. doi: 10.1371/journal.pone.0282507 (PMC9987795; doi:10.1371/journal.pone.0282507)
Supplement: S10 Table — In your opinion, on which things should the rating be based? Please list the three most important things on which to evaluate the quality of these animal facilities (from most to least important)”. (DOCX) [file pone.0282507.s010.docx]

**Table S10.** Distribution of categories identified in visitors answers to question n.19 “Imagine an official five star rating system that rates the quality of the facilities in which animal-visitor interactions take place. In your opinion, on which things should the rating be based? Please list the three most important things on which to evaluate the quality of these animal facilities (from most to least important)”

| **First most important criteria** | | **Second most important criteria** | | **Third most important criteria** | | **Overall most important criteria** | |
| --- | --- | --- | --- | --- | --- | --- | --- |
| **Category** | **% (n)** | **Category** | **% (n)** | **Category** | **% (n)** | **Category** | **% (n)** |
| Animal welfare and care | 70% (121) | Animal welfare and care | 20% (34) | Education | 19% (33) | Animal welfare and care | 34% (179) |
| Cleanliness and Hygiene | 5% (8) | Education | 16% (27) | Staff | 14% (25) | Education | 13% (65) |
| Safety | 5% (8) | Safety | 14% (25) | Animal welfare and care | 14% (24) | Staff | 10% (54) |
| Staff | 3% (6) | Characteristics of the facilities | 10% (17) | Safety | 10% (18) | Safety | 10% (51) |
| AVI characteristics | 3% (5) | AVI characteristics | 7% (12) | Experience quality and service | 8% (13) | Characteristics of the facilities | 5% (25) |
| Education | 3% (5) | Cleanliness and Hygiene | 5% (8) | Ethics, Mission and Sustainability | 6% (10) | Cleanliness and Hygiene | 5% (24) |
| Ethics, Mission and Sustainability | 2% (4) | Experience quality and service | 4% (7) | Cleanliness and Hygiene | 5% (8) | AVI characteristics | 4% (22) |
| Characteristics of the facilities | 1% (1) | Ethics, Mission and Sustainability | 2% (4) | Conservation | 5% (8) | Experience quality and service | 4% (21) |
| Experience quality and service | 1% (1) | Conservation | 1% (2) | Characteristics of the facilities | 4% (7) | Ethics, Mission and Sustainability | 3% (18) |
| Missing answer | 3% (6) | Staff | 13% (23) | AVI characteristics | 3% (5) | Conservation | 2% (10) |
| N/A | 5% (8) | Missing answer | 3% (6) | Missing answer | 8% (13) | Missing answer | 5% (25) |
|  |  | N/A | 5% (8) | N/A | 5% (9) | N/A | 5% (25) |
|  | n=173 |  | n=173 |  | n=173 |  | n=519 |
