# Supplementary material for: Ethical reasoning and participatory approach towards achieving regulatory processes for animal-visitor interactions (AVIs) in South Africa
Source: PLoS One. 2023 Mar 6;18(3):e0282507. doi: 10.1371/journal.pone.0282507 (PMC9987795; doi:10.1371/journal.pone.0282507)
Supplement: S11 Table — (DOCX) [file pone.0282507.s011.docx]

**Table S11.** Answers to questions 4-16, “respect for” principle of the statements, the percentage of respondents choosing each score for each statement, the mean, median, and mode.

| **n.** | **"Please indicate the level of agreement regarding the following statements"** | **Respect for** | **% respondents choosing each score of the five points Likert scale (where 1 = Strongly disagree and 5 = Strongly agree)** | | | | | **Mean [score]** | **Median [score]** | **Mode [score]** |
| --- | --- | --- | --- | --- | --- | --- | --- | --- | --- | --- |
|  |  |  | 1 | 2 | 3 | 4 | 5 |  |  |  |
| 4 | My workplace allows me to balance my work and personal/family life by offering me the support I need when I need it | Wellbeing |  |  | 7,1 | 64,3 | 28,6 | 4,21 | 4,00 | 4,00 |
| 5 | My workplace provides me with the resources, tools and support that I need to do my job to the best of my ability | Autonomy;  Fairness |  |  |  | 64,3 | 35,7 | 4,36 | 4,00 | 4,00 |
| 6 | My workplace provides me with satisfactory income | Wellbeing |  | 7,1 | 21,4 | 64,3 | 7,1 | 3,71 | 4,00 | 4,00 |
| 7 | My workplace environment provides me with satisfactory safety conditions, considering the unique context of my work | Wellbeing |  |  |  | 64,3 | 35,7 | 4,36 | 4,00 | 4,00 |
| 8 | I am able to fully apply my knowledge and skills to my job | Autonomy |  |  |  | 42,9 | 57,1 | 4,57 | 5,00 | 5,00 |
| 9 | My workplace provides me with opportunities for practical training and professional development | Autonomy;  Fairness |  |  | 28,6 | 57,1 | 14,3 | 3,86 | 4,00 | 4,00 |
| 10 | My workplace allows me to be updated on animal welfare, conservation issues, education, and current legislation. | Autonomy;  Fairness |  |  |  | 78,6 | 21,4 | 4,21 | 4,00 | 4,00 |
| 11 | I feel that opportunities and recognition go to those who deserve them | Fairness |  |  | 7,1 | 92,9 |  | 3,93 | 4,00 | 4,00 |
| 12 | My working condition allows me to behave according to the unique relationship I have with the animals I am in care of | Wellbeing |  |  |  | 57,1 | 42,9 | 4,43 | 4,00 | 4,00 |
| 13 | My work environment allows me to feel appreciated and respected | Fairness;  Wellbeing |  |  | 14,3 | 85,7 |  | 3,86 | 4,00 | 4,00 |
| 14 | I have the possibility to provide animals with the attention and resources they need | Autonomy |  |  |  | 57,1 | 42,9 | 4,43 | 4,00 | 4,00 |
| 15 | I feel I had an adequate training to work with animals and visitors during the interactions | Autonomy |  |  |  | 57,1 | 42,9 | 4,43 | 4,00 | 4,00 |
| 16 | I feel my job is protected by an adequate legislation | Fairness |  |  | 14,3 | 71,4 | 14,3 | 4,00 | 4,00 | 4,00 |
| Tot 4-16 | Perceived respect for Staff’s Wellbeing, Autonomy and Fairness |  |  |  |  |  |  | 4,18 | 4,00 | 4,00 |
