# Supplementary material for: Ethical reasoning and participatory approach towards achieving regulatory processes for animal-visitor interactions (AVIs) in South Africa
Source: PLoS One. 2023 Mar 6;18(3):e0282507. doi: 10.1371/journal.pone.0282507 (PMC9987795; doi:10.1371/journal.pone.0282507)
Supplement: S12 Table — (DOCX) [file pone.0282507.s012.docx]

**Table S12**. Suggestions given by respondents in question n. 25

| **Question n. 25: Do you have suggestions on how to improve the Animal-Visitor Interaction activities? Consider whatever in your opinion is relevant (animal welfare, visitor experience, your work and role in it, management, anything else)** |
| --- |
| The media needs to change its perspective and stop painting all facilities with one brush. This would in turn improve public opinion regarding animal interactions, which would allow facilities to generate enough revenue to employ additional staff and cover the running and conservation costs of the animals under their care. Visitors should be encouraged to continue in the support of good facilities by donations and adopt an animal campaign in order to further support. An online presence for most facilities is recommended. |
| Improve the feeding of the farm that the elephants are based on. i.e. more trees/feed for elephants on the farm |
| Conducting interactions in the bush with the elephants. A more natural experience |
| Make sure that the correct safety procedures is practiced for both the guests and the staff. |
