# Supplementary material for: Ethical reasoning and participatory approach towards achieving regulatory processes for animal-visitor interactions (AVIs) in South Africa
Source: PLoS One. 2023 Mar 6;18(3):e0282507. doi: 10.1371/journal.pone.0282507 (PMC9987795; doi:10.1371/journal.pone.0282507)
Supplement: S13 Table — (DOCX) [file pone.0282507.s013.docx]

**Table S13**. Final detailed EM

|  | WELLBEING | AUTONOMY | FAIRNESS |
| --- | --- | --- | --- |
|  | Health & welfare | Freedom & choice | Equity & justice |
|  | *Maximizing the good, minimizing the harm* | *Valuing differences and individual freedom* | *Avoiding discrimination* |
| Animals Involved in AVI | **Animal Welfare**   - Improving health, husbandry, possibility of express species-specific patterns of behaviour, favouring positive interactions with conspecifics, environment, human caregivers (improving stockman ship), and visitors - Living a life worth living - Animal welfare assessment specie-specific - AVI assessment (evaluate effects of AVI on animal welfare)   **Safety**   - Minimizing risks of physical harm and zoonotic diseases transmission - Minimizing risks to cause damage to human caregivers and visitors, as these may lead to negative outcomes also for the animal - Being protected by human treats (i.e. poaching) | **Behavioural freedom**   - expression of normal behavioural patterns - ability to choose not to interact with visitors - having a degree of control on the environment - Being involved in the kind of AVI which brings the better outcome in term of animal welfare   **Right to be captive and right to be rewilded**, according to individual characteristics of the animal and environmental conditions (wild animal population and biodiversity, Human-animal conflict, Poaching…).   - Living a life worth living in the natural habitat or in captivity | **Avoid increasing objectification**, animals = sentient beings  **Equity of treatment**   - Equitable conditions between individuals (i.e., do not overexpose an individual to AVI because it is easier to handle it compared to the others) - Equal entitlement to care - Equal opportunities in terms of wellbeing and positive outcomes   **Respect their role of “ambassador**  **animals**”  **Animal welfare standards not being influenced by human dissents and conflicts** (at facility level or between stakeholders)  **Legal protection**   - being protected by the law |

| Owners And Managers | **Satisfactory working conditions**   - Possibility to work alongside competent and dedicated staff (possibility of being advised and/or constructively criticized, possibility to delegate and share responsibilities) - Being supported on the workplace (responsibility could be taxing) - Stimulating working environment - Having an economically rewarding job - Having a workplace which allows to balance work and personal/family life. Avoid work overload - Personal fulfilment and self-realization   **Sustainability**   - Of their enterprises, with possibility of their positive development - Of the industry - Having access to founds to carry out recognised conservation projects   **Wellbeing of animals, staff and visitors**   - Interest in guaranteeing safety and wellbeing of animals, employers, and visitors under their responsibility (both in its legal and emotional dimension)   **Property interest**   - ownership and/or control of land and resources   **Having support and the approval of society and Institutions** | **Managerial freedom**   - Freedom to use - Being supported terms of space, instruments, skills, and education - Freedom to choose and support in choosing the best management strategies to promote:   - the activities and mission of the facility   - the wellbeing of staff and animals   - biodiversity in their properties   - Conservation education   - Employment opportunities for rural communities - Freedom to innovate - Clear and simple legislation and regulation which promotes facility compliance   **Professional development and support**   - Being provided of opportunities for update and open conversation, possibly in the context of a ‘self-regulating body’ able to understand actual challenges in running an animal facility in SA - Accountable and efficient communication   - With staff   - With other facilities   - With public authorities   - With other stakeholders - Feeling to have an adequate training and k - knowledge to operate   **Recognition of the peculiar features of each facility**   - Possibility to be officially recognised as a facility offering overall high standards (i.e. in terms of animal welfare, conservation, poverty alleviation at a local level, and conservation education) - Stop painting all facilities with one brush | **Fair legislation and regulations** which   - Support sustainability of the enterprise - Avoid favouring some individual - Avoid marginalizing some facilities - Equal possibility to develop and grow - Clarity of the rules to be respected   **Equal possibility to communicate** with public, institutions and between representative bodies; accountable communication process, devoid of unfair accusations  **Fair assessment of the features of the facility**(desert)   - Competent auditors to conduct regular inspections and attest actual standards of the facility, certifying compliance with law requirements - Animal welfare assessment - Conservation efforts assessment - Conservation education assessment   **Fair recognition of the actual/potential role of the facility in fulfilling**  **Conservation and/or Education purposes, along with entertainment opportunities** |
| --- | --- | --- | --- |
| Staff Involved in AVI | **Safety**   - Working in a safe and secure environment, also during the interactions. - Minimizing risks of physical harm and zoonotic diseases.   **Satisfactory working conditions**   - Having an economically rewarding job. - Having a workplace which allows to balance work and personal/family life - Having a personally rewarding job; feeling appreciated and respected as a person and as a professional - Working in a conductive environment (responsibility could be taxing) - Personal fullfilment and self-realization   **Avoid cognitive dissonance**   - Freedom to behave reflecting their personal values - Possibility to behave according to their knowledge about animals and their peculiar relationship with the animals under their care | **Professional freedom**   - Freedom to use their skills and judgement - choose the tasks that best reflect personal skills - Expressing opinions around feasibility of AVI, according to their knowledge and understanding of the animals under their responsibility - Being able to work independently   - in terms of provision of resources and tools   - in terms of education   - in terms of being able to update on the new research about animal welfare, conservation, education, and the current legislation   **Professional development**   - Being provided of opportunities for practical training and professional development - Feeling to have an adequate training to work both with animals and visitors   **Respect for caregivers’ professional ethics**   - Being able to provide animals with the attention and resources they need - Taking part to management strategies to promote the wellbeing of the animals and to contribute to conservation and education missions   **Being able to be compliant with the law** | **Equal opportunities**   - Having equal access to funds to develop and growth (I.e. professional updating and any resources, tools, support) - Being protected at the working place by a clear and adequate legislation - Fair distribution of the risk among the staff members   **Fair staff recognition**   - Recognition go to those who deserve it (desert)   **Respect for caregiver professional role**   - Being appreciated and respected as professionals. Developing effective, accountable communication process with the managers, to empower the staff in expressing their point of view - Being in the conditions to be compliant with the law - Contributing to the fulfilling of the mission statement of the facility |
| Veterinarians | **Safety**   - Working in a safe and secure environment - Minimizing risks of physical harm and zoonotic diseases.   **Satisfactory working conditions**   - Having an economically rewarding job. - Avoiding compassion fatigue and burnout (responsibility could be taxing) - Having the possibility to cooperate with staff and managers in a positive working environment and through transparent communication - Positive feeling in accomplishing their duties/vocation and promoting physiological and psychological welfare for the animals | **Professional freedom**   - Freedom to use their skills, knowledge, and judgement - Being supported with spaces, instruments, founds, training and updating possibilities in order to prevent, diagnosis, treat diseases and pain. - Contributing to choices that impact on the wellbeing of the animals as individuals, groups, and species - In presence of animal welfare or health issues, expressing recommendations around the feasibility of AVI   **Possibility to respect professional ethics**   - Having the possibility to operate to the best of their knowledge and judgment to guarantee animal welfare and protection of animal and human health. More in detail, having the possibility to carry out activities concerning:   - protection of man and animals from the environmental dangers, animal diseases and zoonotic diseases   - prevention, diagnosis, and treatment of animal diseases   - protection of animal welfare   - conservation of environment and fauna heritage, promoting biodiversity and compatible coexistence with human beings.   - endorsing respect for animals as sentient beings   - Being able to work   - Education/promotion on hygienic-health prevention and an healthy human-animal-environment relationship.   - independently (spaces,   - instruments, skills, and   - Possibility to participate in scientific projects   **Being able to be compliant with the law** | **Being respected as professionals** by managers, staff, animal rights groups, governative representative and general public.   - Actual implementation of the veterinarian’s recommendations and treatment prescriptions (when possible) - Being recognised as advocates of animals’ wellbeing   **Equitable standards of practice**  **Fair price for their work** |
| Government representatives | **Development** of the Country  **Personal fulfilment** and self-realization  **Being supported** in their work by cooperative stakeholders and different government departments | **Being educated** and informed   - Avoid misinformation   **Possibility to respect their own institutional role**, free from pressure of single stakeholders (professional ethics)  **Being provided with resources** to accomplish their institutional duties | **Respect of regulations**  **Respect for their institutional role**  **Fair involvement of the different departments** |
| Biodiversity | **Conservation**   - Improving scientific knowledge on species and their habitat - AVI used as a tool to deliver conservation education and inspire people to act with pro-conservation attitude - Animal facilities preventing land conversion to agriculture. I.e. by provision of economic support to maintain the natural integrity of privately-owned ecosystems, which might otherwise be lost through conversion to other forms of land use such as agricultural monocropping (Williams & Sas-Rolfes, 2019)   **Mitigating human -animal conflict through education and poverty alleviation** of the local rural communities | **Autonomy from human intervention**   - Preventing illegal taking of animals from the wild - Preventing poaching - Avoid direct and indirect contact with wild herds   **Availability of sufficient resources** | **Equal respect for each component of Nature**   - Flagship species providing protection also to other species and landscape - Equal distribution of the resources - Being protected by the law - Fair assessment of the conservation projects |
| Visitors | **Safety**   - Actual and perceived. Minimizing risks of physical harm and zoonotic diseases   **Satisfactory experience**   - Amusement - Being entertained - Having a thrilling experience - Enjoying learning - Entertainment and satisfaction provided by other services /experiences offered by the facility (i.e. bush walks, accommodation, hospitality, landscape)   **Possibility to be emotionally close to animals**   - Possibility to empathize with animals. - Experience positive emotional impact resulting from being physically and emotionally close to animals and nature (“closer” form of connection with them).   **Avoid cognitive dissonance**   - Visitors experiencing AVI may feel ‘cognitive dissonance’ associated with doing the experience the animals (and enjoying it) over concern - with animal welfare, mission statements of the facility, origin and destination of the animals (i.e. lion cubs) | **Having the opportunity to see wild animals** in a controlled environment  **Possibility to choose** to interact with animals, be educated, do entertainment activities, do emotional experience, be physically and emotionally close nature and animals  **Education** (During and beyond the AVI)   - Possibility to have back ground information, receiving conservation education thanks to national education system - Avoid misinformation - Contextually to AVI, having the opportunity to:   - Learn about animals (anatomy, physiology, ethology, captive animal welfare, husbandry, management, keeper-animal relationship, handler-animal relationship, training).   - Learn about habitats and biodiversity; understand the interrelationship between wildlife and environments; learn about Interdependence.   - Learn about conservation (rehabilitation, reintroduction, species survival plan, current challenges, poaching, conservation sustainability, understand man's impact)   - Inspire ethical reasoning on AVIs and conservation. Encourage questions about:     - origin, history and life of the animal housed in the facility (why these animals are here?)     - mission statements of the facility     - purpose of AVIs itself     - facility activities (rescue centre, educational programs for vets, students etc, worldwide partnerships and cooperation)   **Informed consent**  Have the freedom to choose a facility and an AVIs being informed about relevant aspects (i.e. knowing its "animal welfare standards", details of the interactions, facility mission statements, risks etc.) | **Affordability**   - Fair price   **Accessibility**   - Equal opportunities to access to information about the facility, its mission statements, AVI   **Equal opportunities concerning**:   - Education and information - access to natural resources and to be close to nature and animals (physically and emotionally) - right to benefit from the mission of the facility (animal welfare, conservation, education) |
| Animal Rights Groups | **Sustainability of their business**   - Provision of founds to keep operating (i.e. donations)   **Personal fulfilment** and self-realization of their members | **Freedom to propose their long-term vision** on SA tourism industry with regard to AVI  **Freedom to communicate their ideas** regarding AVI  **Education** and access to information, avoiding miseducation | **Being recognised as a group of people advocating their own perspective on AVI**  **Equal access to communication** |
